# Effectiveness and safety of pembrolizumab, nivolumab, and atezolizumab as adjuvant therapy for high-risk muscle-invasive urothelial carcinoma: an indirect comparison

**DOI:** 10.3389/fonc.2024.1527540

**Published:** 2025-01-23

**Authors:** Wei Chen, Soichiro Yoshida, Noriyoshi Miura, Shohei Fukuda, Hiroshi Fukushima, Yuma Waseda, Hajime Tanaka, Yasuhisa Fujii

**Affiliations:** ^1^ Department of Urology, Institute of Science Tokyo, Tokyo, Japan; ^2^ Department of Urology, Zigong Fourth People’s Hospital, Zigong, Sichuan, China; ^3^ Department of Urology, Ehime University, Matsuyama, Japan

**Keywords:** adjuvant immunotherapy, immune checkpoint inhibitor, muscle-invasive urothelial carcinoma, PD-1/PD-L1 inhibitor, Shiny method

## Abstract

**Background:**

The effectiveness of immune checkpoint inhibitors (ICIs) as adjuvant therapy for muscle-invasive urothelial carcinoma (MIUC) with high recurrence risk has been demonstrated. With no direct efficacy comparisons available, we aimed to indirectly compare the efficacy and safety of pembrolizumab, nivolumab, and atezolizumab as adjuvant treatments for high-risk MIUC based on individual patient data (IPD) from clinical trials.

**Methods:**

IPD was reconstructed using the Shiny method from Kaplan–Meier curves of eligible randomized controlled trials. We compared disease-free survival (DFS), overall survival (OS), PD-L1 positive DFS between treatments, and assessed treatment-related adverse events (TRAE).

**Results:**

Four studies including 2,220 high-risk MIUC patients showed no statistically significant difference between the three agents in terms of DFS (pembrolizumab vs. nivolumab: HR 0.97, 95% CI 0.79–1.18; pembrolizumab vs. atezolizumab: HR 0.85, 95% CI 0.70–1.04; nivolumab vs. atezolizumab: HR 0.90, 95% CI 0.74–1.10). All three agents showed comparable DFS outcomes in PD-L1 positive patients (pembrolizumab vs. nivolumab: HR 1.16, 95% CI 0.83–1.60; pembrolizumab vs. atezolizumab: HR 0.85, 95% CI 0.84–1.14; nivolumab vs. atezolizumab: HR 0.79, 95% CI 0.57–1.09), with similar DFS rates 24- and 36-months post-treatment (pembrolizumab: 53.3% and 46.8%; nivolumab: 48.5% and 44.8%; Atezolizumab: 45.0% and 40.7%). OS data showed no significant differences between pembrolizumab and nivolumab (HR 1.16, 95% CI: 0.90–1.49), pembrolizumab and atezolizumab (HR 1.02, 95% CI: 0.81-1.30), and nivolumab and atezolizumab (HR 0.87, 95% CI: 0.69–1.09). TRAE incidence varied but remained manageable (any grade: 26.4% pembrolizumab, 78.6% nivolumab, 54% atezolizumab; grade ≥3: 21.8% pembrolizumab, 18.2% nivolumab, 16.0% atezolizumab).

**Conclusions:**

All three agents showed similar efficacy with manageable safety profiles, positioning them as promising adjuvant therapies for MIUC. These results provide an evidence-based framework for clinical decision-making despite the lack of direct comparative data.

## 1 Introduction

Despite advancements in treatment, management of muscle-invasive urothelial carcinoma (MIUC) continues to be a significant challenge, as approximately one-quarter of newly diagnosed bladder cancers presents as muscle-invasive disease (1). However, even with improved surgical techniques and perioperative management, the 5-year overall survival (OS) rate for patients with muscle-invasive bladder cancer after radical cystectomy is approximately 50–65% (2), which suggests the need for more effective treatment strategies to promote long-term outcomes.

The standard of care for MIUC has been neoadjuvant platinum-based chemotherapy (3). Nevertheless, a significant proportion of patients fail to receive neoadjuvant chemotherapy or their disease relapses despite this approach. Recently, immune checkpoint inhibitors (ICIs) have revolutionized the treatment landscape for several cancers including urothelial carcinoma (4). However, recent clinical trials of ICIs in adjuvant therapy for MIUC have shown inconsistent results (5–8). In the CheckMate 274 trial, nivolumab was found to significantly improve disease-free survival (DFS) (6), while the IMvigor010 trial with atezolizumab did not achieve its primary DFS endpoint (7). A new study suggested that pembrolizumab improved DFS, but it was unclear whether it would have a significant impact on OS (5). These conflicting results have led to only limited recommendations for adjuvant ICI therapy in current guidelines (9).

Considering these varying outcomes and the lack of direct comparative data between pembrolizumab, nivolumab, and atezolizumab for adjuvant treatment of MIUC, clinicians may have difficulty selecting the appropriate adjuvant immunotherapy. Hence, we conducted a study with individual patient data (IPD) from clinical trials to indirectly compare the efficacy and safety between pembrolizumab, nivolumab and atezolizumab as adjuvant therapy for MIUC, to address these issues and to provide guidance for clinical decision-making.

## 2 Methods

### 2.1 Literature identification and selection

We conducted a comprehensive literature search using databases including PubMed, Embase, Clinicaltrials, and the Cochrane Central Register of Controlled Trials. The search period was from the inception of the database to September 26, 2024. We used a combination of Medical Subject Headings (MeSH) terms and free text words associated with MIUC, adjuvant therapy, and immune checkpoint inhibitors. The search strategy was urinary bladder neoplasms, carcinoma, transitional cell, muscle-invasive urothelial carcinoma, MIUC, urothelial cancer, and bladder cancer for disease; immunotherapy, ICIs, programmed cell death-1 (PD-1) inhibitors, programmed cell death ligand-1 (PD-L1) inhibitors, pembrolizumab, nivolumab, atezolizumab, and adjuvant immunotherapy for treatments; and clinical trial, meta-analysis, systematic review for publication type. We followed the Preferred Reporting Items for Systematic Reviews and Meta-analyses (PRISMA) guidelines for literature screening.

The inclusion criteria were: (a) randomized controlled trials; (b) studies of pembrolizumab, nivolumab, or atezolizumab as adjuvant therapy of patients with MIUC; (c) studies providing Kaplan–Meier (KM) curves and risk tables for outcomes of interest; and (d) studies published in English. The exclusion criteria were: (a) non-randomized studies; (b) studies without relevant outcome data; and (c) duplicate publications or secondary analyses of included trials.

### 2.2 Data extraction and quality assessment

The titles and abstracts of the retrieved records were independently screened by two investigators. Potentially eligible studies were full text reviewed. A preset form was used to collect data, including study design, basic characteristics, intervention details and outcomes. The original data was used to resolve all disagreements through discussion. The main outcome of interest was disease-free survival (DFS). We also studied overall survival (OS) and treatment-related adverse events (TRAE) as additional important outcomes. The included studies were assessed using the Cochrane Risk of Bias tool 2.0 v9, including the randomization process, deviations from intended interventions, missing outcome data, outcome measurement, and selective reporting of results.

### 2.3 Reconstruction and validation of IPD

The coordinates of each curve included in the study were primarily extracted from the original KM curve using Digitize software (FSF, Inc., Boston, USA). IPD was reconstructed from these coordinates and corresponding risk tables by the *IPDfromKM* package introduced by Liu et al. in 2021 (10). This reconstruction process involved digitizing survival curves to obtain time and survival probability coordinates. The algorithm used published risk tables showing the number of patients at risk at different time points to estimate the timing of events and censoring, producing survival curves that matched the original data. To validate the reconstructed data, we plotted KM curves using the reconstructed IPD and compared them with the original KM curves for qualitative evaluation. Moreover, quantitative validation was further performed by comparing the hazard ratio (HR) and the 95% confidence interval (CI) calculated from the reconstructed data with the values previously reported in the original publications.

### 2.4 Statistical analysis

A Cox proportional hazards model was applied to estimate HR with 95% CI to test the main hypothesis. For treatment comparisons across different trials, we used the reconstructed IPD to perform pairwise comparisons between treatment arms. The results were then compared with the original dataset using a Z-test to assess variation. We combined the reconstructed IPD from treatment and control arms of each study into a unified dataset. Using this combined dataset, we generated survival curves for DFS and OS across the three treatment groups and their respective control arms. The 24-month and 36-month DFS and OS rates were calculated for each group. A subgroup analysis was conducted to determine the outcome of DFS on PD-L1-positive patients. Statistical analyses were performed in R version 4.0.3 (R Foundation for Statistical Computing, Vienna, Austria), and significance was defined as a two-sided p-value less than 0.05.

## 3 Results

### 3.1 Basic characteristics of study populations

**Figure 1:**
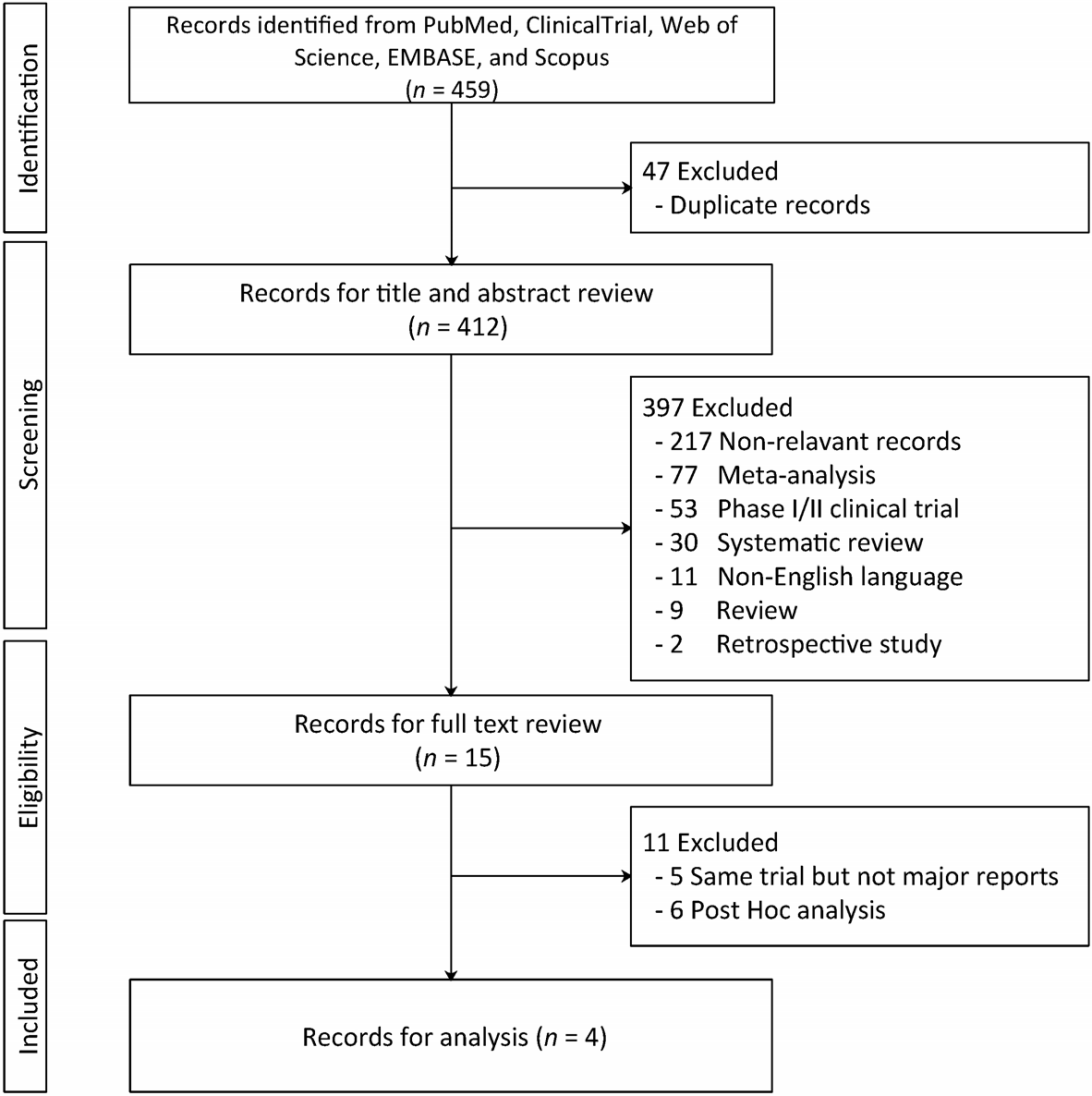
Preferred Reporting Items for Systematic Reviews and Meta-analyses (PRISMA) diagram.

In total, four studies (5, 7, 8, 11) from three trials (AMBASSADOR, CheckMate 274, and IMvigor010) composing 2,220 patients in three trials were included after screening in accordance with the PRISMA diagram (**Figure 1**). A similar set of entry criteria was identified in all three studies that focused on patients with high-risk MIUC following radical surgery and candidates for adjuvant therapy. **Table 1** shows the basic characteristics of the included trials. The age of participants ranged from a median of 65.3 to 69 years. Male patients comprised 72.70–79.31% of the populations in all studies. Racial distribution was also quite variable, with AMBASSADOR reaching 89.08–91.24% White patients, while CheckMate 274 and IMvigor010 comprised 74.79–78.82% White patients. However, Asian patients comprised a larger proportion of the IMvigor010 (15.76–16.87%) and CheckMate 274 (21.07–22.66%) than AMBASSADOR (1.41–2.87%). Most patients across all studies had an Eastern Cooperative Oncology Group (ECOG) performance status of 0 or 1. The proportion of patients with an ECOG status of 0 was higher in AMBASSADOR (51.44–51.98%) than CheckMate 274 and IMvigor010 (61.08–64.27%). Bladder was the predominant primary tumor site in all studies, while the proportion was varied. Specifically, the range of bladder cancer was 75.42–79.04% in AMBASSADOR and CheckMate 274, but 92.86–93.80% in IMvigor010. The positive surgical margin proportion in AMBASSADOR trial was 2.42% (17/702), whereas such cases were not included in the CheckMate 274 and IMvigor010 trials. The number of lymph nodes resected was similar between CheckMate 274 and IMvigor010, but this information was not reported for the AMBASSADOR. All trials allowed variant histology with a dominant urothelial carcinoma pattern. The proportion of patients with variant histology varied from 9.97% (70/702) in AMBASSADOR to 40.34% (286/709) in CheckMate 274, while IMvigor010 did not report the detail data. Most trials had a low risk of bias, as shown in **Supplementary File 1**. Validation of the IPD is shown in **Supplementary File 2**, which suggested the reliability and accuracy of the reconstructed data.

**Table 1:** Basic characteristics of included trials.

Characteristic	Treatment arm			Control arm		
	AMBASSADOR	CheckMate 274	IMvigor010	AMBASSADOR	CheckMate 274	IMvigor010
Design	Pembrolizumab	Nivolumab	Atezolizumab	Observation	Placebo	Observation
Sample Size	n = 354	n = 353	n = 406	n = 348	n = 356	n = 403
Age - Median (range)	69 (22–92)	65.3 (30–92)	67 (60–72)	68 (34–90)	65.9 (42–88)	66 (60–73)
Sex						
Male	271 (76.55%)	265 (75.07%)	322 (79.31%)	253 (72.70%)	275 (77.25%)	316 (78.41%)
Female	83 (23.45%)	88 (24.93%)	84 (20.69%)	95 (27.30%)	81 (22.75%)	87 (21.59%)
Race						
White	323 (91.24%)	264 (74.79%)	320 (78.82%)	310 (89.08%)	272 (76.40%)	307 (76.18%)
Asian	5 (1.41%)	80 (22.66%)	64 (15.76%)	10 (2.87%)	75 (21.07%)	68 (16.87%)
Black	14 (3.95%)	2 (0.57%)	3 (0.74%)	11 (3.16%)	3 (0.84%)	3 (0.74%)
Other/Unknown	12 (3.39%)	7 (1.98%)	19 (4.68%)	17 (4.89%)	6 (1.69%)	25 (6.20%)
ECOG Performance Status						
0	184 (51.98%)	224 (63.46%)	248 (61.08%)	179 (51.44%)	221 (62.08%)	259 (64.27%)
1	151 (42.66%)	122 (34.56%)	142 (34.98%)	157 (45.11%)	125 (35.11%)	130 (32.26%)
2	19 (5.37%)	7 (1.98%)	16 (3.94%)	12 (3.45%)	9 (2.53%)	14 (3.47%)
Primary Tumor Site						
Bladder	267 (75.42%)	279 (79.04%)	377 (92.86%)	263 (75.57%)	281 (78.93%)	378 (93.80%)
Upper Tract	81 (22.88%)	74 (20.96%)	29 (7.14%)	73 (20.98%)	75 (21.07%)	25 (6.20%)
Pathological Tumor Stage						
≤pT2	189 (53.39%)†	87 (24.65%)	104 (25.62%)	178 (51.15%)†	94 (26.40%)	101 (25.06%)
pT3 or pT4	165 (46.61%)‡	263 (74.50%)	302 (74.38%)	170 (48.85%)‡	266 (74.72%)	302 (74.94%)
Histology variant	38 (10.73%)	145 (41.08%)	NR	32 (9.20%)	141 (39.61%)	NR
Positive surgical margins	9 (2.50%)	0 (0.00%)	0 (0.00%)	8 (2.30%)	0 (0.00%)	0 (0.00%)
Number of lymph nodes resected (<10)	NR	94 (26.60%)	95 (23%)	NR	99 (27.80%)	94 (23.00%)
Pathological Nodal Status						
Positive	180 (50.85%)	167 (47.31%)	212 (52.22%)	170 (48.85%)	168 (47.19%)	208 (51.61%)
Negative	174 (49.15%)	185 (52.41%)	194 (47.78%)	178 (51.15%)	187 (52.53%)	195 (48.39%)
Previous Neoadjuvant Therapy						
Yes	229 (64.69%)	153 (43.34%)	196 (48.28%)	218 (62.64%)	155 (43.54%)	189 (46.90%)
No	125 (35.31%)	200 (56.66%)	210 (51.72%)	130 (37.36%)	201 (56.46%)	214 (53.10%)
PD-L1 Status						
Positive	203 (57.34%)§	140 (39.66%)‡	196 (48.28%)††	201 (57.76%)§	142 (39.89%)¶	196 (48.64%)††
Negative	151 (42.66%)‡	213 (60.34%)‡	210 (51.72%)††	147 (42.24%)§	214 (60.11%)¶	207 (51.36%)††

NR, not reported.

†Positive surgical margins, Any pT, and any N+ are included.

‡pT2 is included.

§PD-L1 positivity criteria not specified in the provided data.

¶PD-L1 positivity defined as ≥1%.

††PD-L1 positivity defined as IC2 or IC3.

### 3.2 Disease-free survival

**Figure 2:**
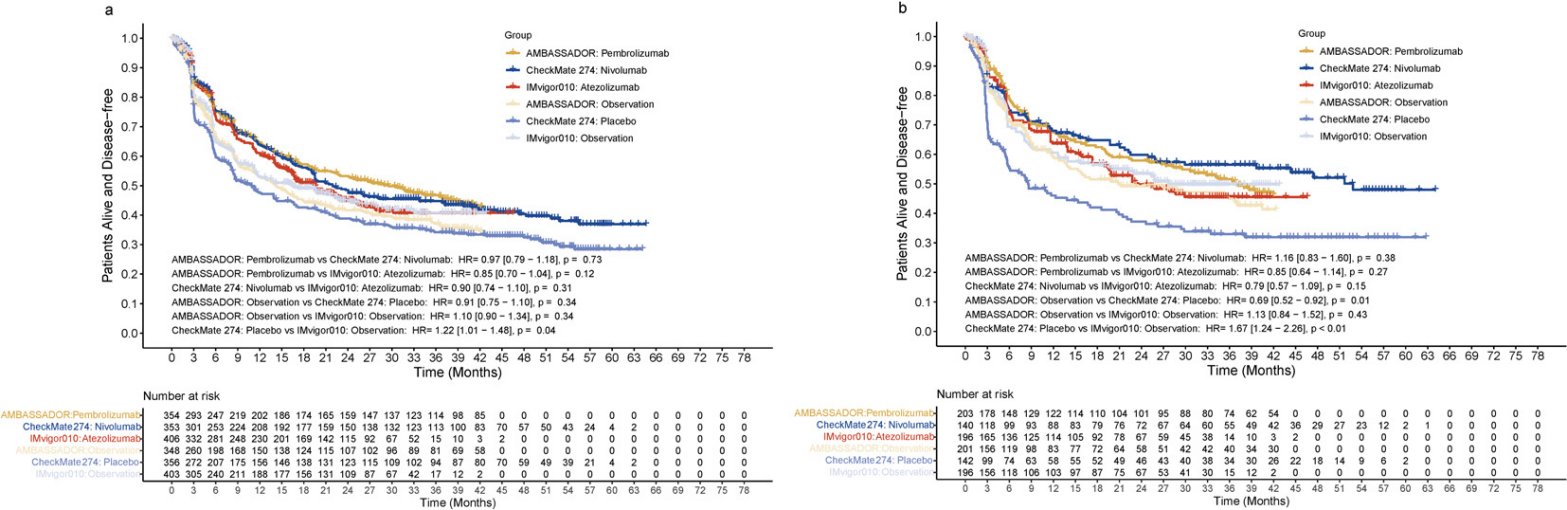
Disease-free survival in the entire cohort **(A)** or in patients positive for PD-L1 **(B)**. HR, hazard ratio; PD-L1, programmed death ligand 1.

All three immunotherapy agents were associated with improved DFS compared with their control arms in the overall population. At the 24-month and 36-month follow-ups, the DFS rates were 53.3% and 46.8% for pembrolizumab, 48.5% and 44.8% for nivolumab, and 45.0% and 40.7% for atezolizumab, respectively. The difference between pembrolizumab, nivolumab and atezolizumab was not significant. The HR for DFS was 0.97 (95% CI: 0.79–1.18, *p* = 0.73) for pembrolizumab versus nivolumab, 0.85 (95% CI: 0.70–1.04, *p* = 0.12) for pembrolizumab versus atezolizumab, and 0.90 (95% CI: 0.74–1.10, *p* = 0.31) for nivolumab versus atezolizumab (**Figure 2A**). Analogous findings were also observed for patients with positive PD-L1 status. The DFS outcomes were comparable among the three agents, with a HR of 1.16 (95% CI: 0.83–1.60, *p* = 0.38) for pembrolizumab versus nivolumab, 0.85 (95% CI: 0.64–1.14, *p* = 0.27) for pembrolizumab versus atezolizumab, and 0.79 (95% CI: 0.57–1.09, *p* = 0.15) for nivolumab versus atezolizumab (**Figure 2B**). Conversely, the control arm in CheckMate 274 showed a significantly worse DFS when compared with that in IMvigor010 (HR=1.22, 95% CI: 1.01–1.48, *p* = 0.04) (**Figure 2A**). The differences in DFS for the control arms were more notable in the subgroup with positive PD-L1 status. DFS in the control arm of CheckMate 274 was significantly worse than that of AMBASSADOR or IMvigor010 (HR=0.69, 95% CI: 0.52–0.92, *p* = 0.01 and <0.01, respectively).

### 3.3 Overall survival

**Figure 3:**
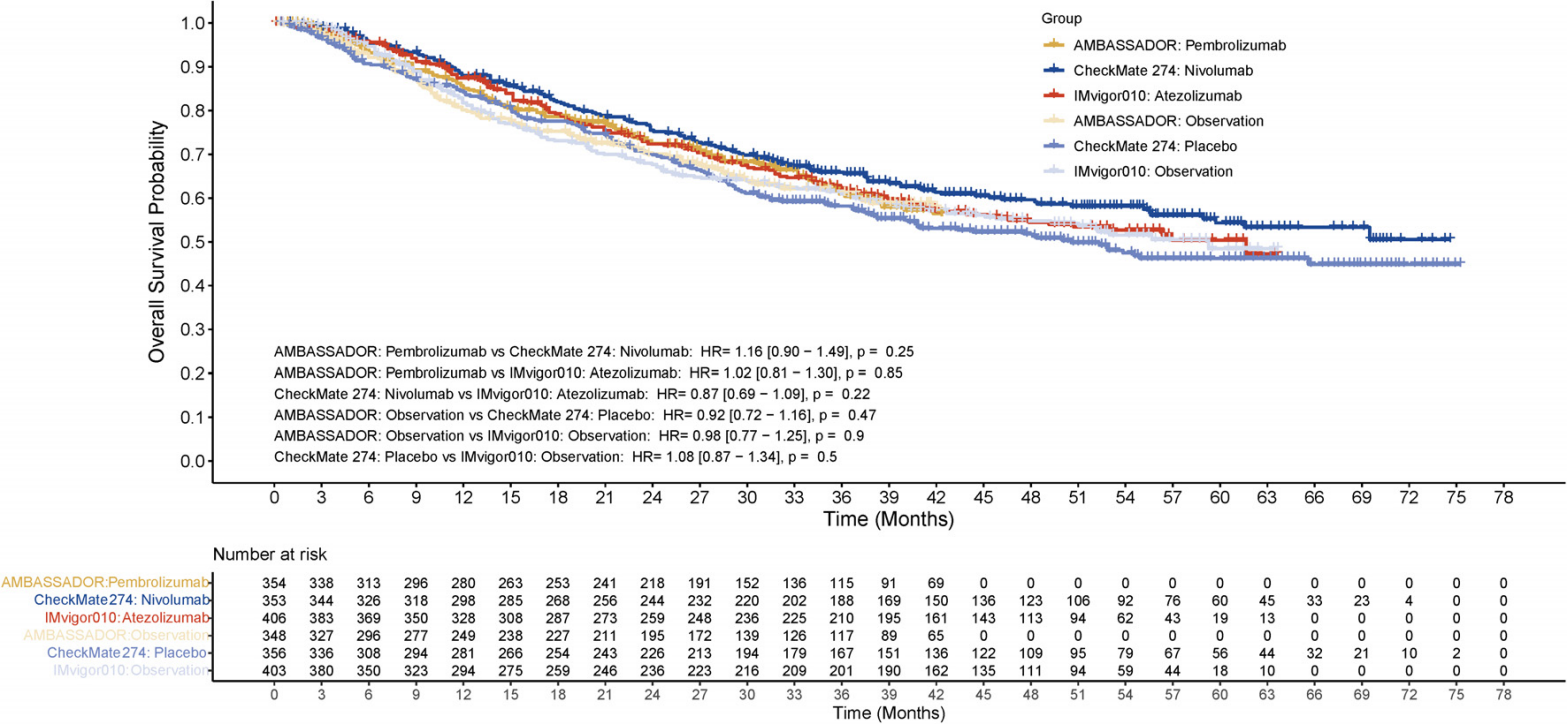
Overall survival in the entire cohort. HR, hazard ratio.

The cross-comparison between these agents revealed no statistically significant differences. Specifically, pembrolizumab versus nivolumab showed an HR of 1.16 (95% CI: 0.90-1.49, *p* = 0.25), pembrolizumab versus atezolizumab had an HR of 1.02 (95% CI: 0.81-1.30, p = 0.85), and nivolumab versus atezolizumab demonstrated an HR of 0.87 (95% CI: 0.69-1.09, *p* = 0.22). All three immunotherapies showed a trend toward improved survival compared to their respective control arms, though the differences did not reach statistical significance. The comparison between control arms showed similar outcomes. Nivolumab appeared to maintain a slightly higher survival probability after 36 months compared to the other treatments, though this difference was not statistically significant. The survival rates were around 72.5% for pembrolizumab, 75.5% for nivolumab, and 72.4% for atezolizumab at 24 months, and 60.9%, 65.9%, and 62.3% at 36 months, respectively (**Figure 3**). Subgroup analysis for positive PD-L1 status was not available for OS at the time of this analysis.

### 3.4 Treatment-related adverse events

Overall, the safety profiles of all three immunotherapies were manageable with some variation in the incidence and type of TRAE (**Table 2**). The incidence of any-grade TRAE was 26.4% in patients receiving pembrolizumab, 78.6% in nivolumab, and 54% in atezolizumab. Grade 3 or higher TRAEs were reported in 21.8%, 18.2%, and 16.0% of patients receiving pembrolizumab, nivolumab and atezolizumab, respectively. The most frequent any-grade TRAE across all three agents were fatigue (47.0% for pembrolizumab, 18.1% for nivolumab, and 16.0% for atezolizumab), pruritus (20–23% across all agents), and diarrhea (21% for pembrolizumab, 17.1% for nivolumab, 9% for atezolizumab). Pembrolizumab was notably more commonly associated with hypothyroidism than nivolumab (20% vs. 10%). For TRAE of grade 3 or higher, pembrolizumab most frequently caused increased lipase (3%), diarrhea (3%), and fatigue (2%). For nivolumab, the most frequent events were pneumonitis (0.9%), colitis (0.9%) and diarrhea (0.9%). In addition, colitis, arthralgia, and increased alanine transaminase (ALT) (all 1%) most often resulted from atezolizumab administration. For patients receiving nivolumab, 12.8% discontinued treatment mostly due to pneumonitis (1.7%). No discontinuation rates were reported for pembrolizumab or atezolizumab in the available data.

**Table 2:** Treatment-related adverse events in each group.

Adverse event†	Any grade			Grade ≥3		
	Pembrolizumab (*n* = 330)	Nivolumab (*n* = 351)	Atezolizumab (*n* = 390)	Pembrolizumab (*n* = 330)	Nivolumab (*n* = 351)	Atezolizumab (*n* = 390)
Total	87 (26.4%)	276 (78.6%)	212 (54%)	72 (21.8%)	64 (18.2%)	64 (16%)
Fatigue	156 (47%)	50 (18.1%)	62 (16%)	8 (2%)	1 (0.3%)	1 (<1%)
Pruritus	74 (22%)	82 (23.4%)	73 (19%)	3 (1%)	0 (0.0%)	2 (1%)
Diarrhea	68 (21%)	60 (17.1%)	34 (9%)	10 (3%)	3 (0.9%)	3 (1%)
Hypothyroidism	66 (20%)	35 (10.0%)	–	0 (0.0%)	0 (0.0%)	–
Arthralgia	59 (18%)	–	22 (6%)	3 (1%)	–	5 (1%)
Rash‡	–	52 (14.8%)	32 (8%)	–	2 (0.6%)	1 (<1%)
Nausea	53 (16%)	–	–	2 (1%)	–	–
Anemia	46 (14%)	–	7 (2%)	5 (2%)	–	2 (1%)
Asthenia	–	–	20 (5%)	–	–	3 (1%)
Pyrexia	–	–	21 (5%)	–	–	2 (1%)
ALT increased	–	15 (4.3%)	14 (4%)	–	2 (0.6%)	4 (1%)
AST increased	–	13 (3.7%)	12 (3%)	–	1 (0.3%)	2 (1%)
Pneumonitis	–	16 (4.6%)	4 (1%)	–	3 (0.9%)	2 (1%)
Colitis	–	7 (2.0%)	1 (<1%)	–	3 (0.9%)	4 (1%)
Lipase increased	42 (13%)	36 (10.3%)	2 (1%)	11 (3%)	18 (5.1%)	3 (<1%)
Acute kidney injury	–	3 (0.9%)	1 (<1%)	–	2 (0.6%)	2 (1%)
Hyperglycemia	19 (6%)	–	–	2 (1%)	–	–
Hyperthyroidism	26 (8%)	33 (9.4%)	–	0 (0.0%)	0 (0.0%)	–

†Events reported between the first dose and 30 days after the last dose of treatment are shown.

‡For pembrolizumab and atezolizumab, “Rash” is listed as “Maculopapular rash”.

ALT increased, AST increased, Pneumonitis, Colitis, Acute kidney, and Hyperthyroidism were observed by the previous CheckMate 274 findings reported Bajorin et al. (6).

## 4 Discussion

From 2017 to 2021, the United States Food and Drug Administration (US FDA) approved these agents for the management of MIUC (12–14). Our indirect comparison using IPD showed that there were no significant differences in efficacy, including DFS and OS, among pembrolizumab, nivolumab, and atezolizumab for the adjuvant treatment of MIUC. The comparable DFS outcomes observed among three immunotherapy agents are particularly encouraging, which suggests that clinicians can choose from several effective adjuvant immunotherapeutic options, and hence can deliver personalized therapy based on patient needs and individual characteristics.

A notable finding from our analysis was the significantly worse DFS in the placebo-controlled arm of CheckMate 274 compared with the observation-only arm in IMvigor010. This difference was particularly pronounced in PD-L1-positive patients, where the CheckMate 274 placebo arm showed significant inferior outcomes compared to both AMBASSADOR and IMvigor010 observation arms. However, these DFS differences did not translate into OS disparities, suggesting that control arm design may primarily influence early outcomes and recurrence patterns rather than long-term survival. The potential trend of DFS favoring pembrolizumab over nivolumab after a 21-month follow-up period, although not statistically significant, warrants careful consideration. However, the observation nature may be significantly influenced by a reduced number of patients in long-term follow-up, particularly after a 27-month follow-up period, which will amplify individual variation and produce statistical bias. Other possible reasons for the trend include differences in immune response kinetics, study designs (In AMBASSADOR and IMvigor010, the control groups did not receive placebo treatment, but were only observed), and patient characteristics. Well-designed head-to-head trials with extended follow-up are needed to determine long-term differences in efficacy between immunotherapies in adjuvant MIUC treatment. Even though PD-L1 expressions may still have prognostic value, our analysis found that among MIUC patients with positive PD-L1 status, pembrolizumab did not have a significant advantage over nivolumab. This result may limit its utility in guiding adjuvant immunotherapy decisions for MIUC. Apolo (5) also suggested that routine PD-L1 testing may not be necessary across all MIUC patients who were receiving adjuvant immunotherapy, but did not guarantee that the results still provided significant utility.

Even though the efficacy of pembrolizumab, nivolumab, and atezolizumab is comparable in the adjuvant therapy for MIUC, the potential differences in safety may contribute to the treatment decisions. All three therapies showed manageable toxicity, but the incidence and types of TRAE differed, suggesting the opportunity to optimize treatment by considering individual patient risk factors and comorbidities. With reference to hypothyroidism, pembrolizumab may also affect decision-making in patients who have underlying thyroid issues with the report in other cancers (15, 16). Also, rates for grade 3 or worse TRAE with pembrolizumab were slightly higher than that with nivolumab and atezolizumab, and specifically increased lipase and diarrhea, may be a consideration for patients who have a history of pancreatitis or inflammatory bowel disease (17). Compared with pembrolizumab, the lower incidence of TRAE in general with nivolumab and atezolizumab would make it an attractive alternative for more frail patients with multiple comorbidities. However, this result should be considered rigorously as the indirect comparison nature might lead to potential bias when comparing the adverse effects originally.

There are several limitations when considering the result of this study. First, the indirect comparison nature was based on case studies rather than head-to-head trials, and so the strength of our conclusions is inherently reduced. To minimize the bias, we compared the reconstructed data with the original data and found the consistency between the two datasets. It is worth noting that the proportion of bladder cancer in IMvigor010 was higher than in the other two trials, which may have affected the efficacy of adjuvant immunotherapy in ways that were not fully accounted for in our analysis. Second, the inconsistency in control group designs across studies is an important constraint. Observational control both in IMvigor010 and AMBASSADOR may have allowed greater flexibility in treatment modifications during follow-up compared to the placebo-controlled design in CheckMate 274. This difference in control arm design may have contributed to the relatively worse DFS outcomes observed in the CheckMate 274 placebo arm, particularly among PD-L1-positive patients who might have had more opportunities to transition to alternative treatments in the observation-only arms. These differences in control group design may partially explain the conflicting results observed in the original studies. Third, the different time points for DFS and OS in the IMvigor010 study should also be noted. The OS data reported by Bellmunt (7) were limited to 48 months of follow-up in 2021, while we selected the latest OS data reported in 2024 by Powles (8). However, the number of patients at risk remained inconsistent after 15 months of follow-up, which meant there might be some adjustments for both the atezolizumab group and the observational group in the extended follow-up results. Moreover, consistent definitions between studies for subgroup analysis, including PD-L1, and the lack of OS data for PD-L1-positive patients were constraints in subgroup analyses. AMBASSADOR used a combined positive score of 10 or higher, while CheckMate 274 focused on PD-L1 staining on at least 1% of tumor cells, and in IMvigor010 the PD-L1 expressing tumor-infiltrating immune cells covering ≥5% of the tumor area was defined as IC2/3. These different methods could have impacted how we interpreted and compared PD-L1-related results between the studies. Finally, the heterogeneity of basic characteristics including variant histology proportion, surgical margins status, and number of lymph nodes resection across trials may limit the evidence of results. However, it should be noted that the classification and diagnosis thresholds of variant subtypes between trials remained significant difference, which could lead to potential heterogeneities. In the absence of uniform standards, we believe this comparison may provide useful information and prompt a more standardized approach in future studies, even though the definitions remain inconsistent. Finally, the quality of our safety profile comparisons may have been affected by the presence of potential inconsistencies in adverse event reporting standards across trials.

In conclusion, our study identified that adjuvant immunotherapy with pembrolizumab, nivolumab, or atezolizumab showed comparable efficacy and safety in patients with MIUC, without significant differences between the agents. These results strengthen adjuvant immunotherapy as a promising option for MIUC treatment. As more long-term data becomes available and prognostic biomarkers are better understood, management strategies will be further refined and, potentially, more personalized treatment will be provided.

## Data Availability

The original contributions presented in the study are included in the article/**Supplementary Material**. Further inquiries can be directed to the corresponding author.

## Supplementary material

Supplementary File 1 Risk of bias assessment according to the Cochrane Collaboration tool RoB2 v9. **(A)** Risk of bias graph that illustrates the proportions of results at low risk, some concerns or high risk; **(B)** Traffic light plot shows the domain and overall judgments study-by-study.

## References

[1] Ghandour R Singla N Lotan Y. Treatment options and outcomes in nonmetastatic muscle invasive bladder cancer. Trends Cancer. (2019) 5:426–39. doi: 10.1016/j.trecan.2019.05.011 31311657

[2] Mak RH Hunt D Shipley WU Efstathiou JA Tester WJ Hagan MP. Long-term outcomes in patients with muscle-invasive bladder cancer after selective bladder-preserving combined-modality therapy: A pooled analysis of radiation therapy oncology group protocols 8802, 8903, 9506, 9706, 9906, and 0233. J Clin Oncol. (2014) 32:3801–9. doi: 10.1200/JCO.2014.57.5548 PMC4239302 25366678

[3] van der Heijden MS Sonpavde G Powles T Necchi A Burotto M Schenker M. Nivolumab plus gemcitabine-cisplatin in advanced urothelial carcinoma. N Engl J Med. (2023) 389:1778–89. doi: 10.1056/NEJMoa2309863 PMC12314471 37870949

[4] Zhu A Garcia JA Faltas B Grivas P Barata P Shoag JE. Immune checkpoint inhibitors and long-term survival of patients with metastatic urothelial cancer. JAMA Netw Open. (2023) 6:e237444. doi: 10.1001/jamanetworkopen.2023.7444 37043205 PMC10098944

[5] Apolo AB Ballman KV Sonpavde G Berg S Kim WY Parikh R. Adjuvant pembrolizumab versus observation in muscle-invasive urothelial carcinoma. N Engl J Med. (2025) 392:45–55. doi: 10.1056/NEJMoa2401726 PMC11698643 39282902

[6] Bajorin DF Witjes JA Gschwend JE Schenker M Valderrama BP Tomita Y. Adjuvant nivolumab versus placebo in muscle-invasive urothelial carcinoma. N Engl J Med. (2021) 384:2102–14. doi: 10.1056/NEJMoa2034442 PMC8215888 34077643

[7] Bellmunt J Hussain M Gschwend JE Albers P Oudard S Castellano D. Adjuvant atezolizumab versus observation in muscle-invasive urothelial carcinoma (imvigor010): A multicentre, open-label, randomised, phase 3 trial. Lancet Oncol. (2021) 22:525–37. doi: 10.1016/S1470-2045(21)00004-8 PMC8495594 33721560

[8] Powles T Assaf ZJ Degaonkar V Grivas P Hussain M Oudard S. Updated overall survival by circulating tumor DNA status from the phase 3 imvigor010 trial: Adjuvant atezolizumab versus observation in muscle-invasive urothelial carcinoma. Eur urol. (2024) 85:114–22. doi: 10.1016/j.eururo.2023.06.007 37500339

[9] EAU-Guidelines-Edn. Presented at the eau annual congress milan 2023. Milan: European Association of Urology (2023).

[10] Liu N Zhou Y Lee JJ. Ipdfromkm: Reconstruct individual patient data from published kaplan-meier survival curves. BMC Med Res Methodol. (2021) 21:111. doi: 10.1186/s12874-021-01308-8 34074267 PMC8168323

[11] Galsky MD Witjes JA Gschwend JE Milowsky MI Schenker M Valderrama BP. Adjuvant nivolumab in high-risk muscle-invasive urothelial carcinoma: Expanded efficacy from checkmate 274. J Clin Oncol. (2025) 43:15–21. doi: 10.1200/JCO.24.00340 39393026 PMC11687940

[12] Food and Drug Administration. FDA approves nivolumab for adjuvant treatment of urothelial carcinoma. (2021). Available online at: https://www.accessdata.fda.gov/drugsatfda_docs/label/2021/125554s097lbl.pdf (Accessed February 1, 2022).

[13] Food and Drug Administration. Pembrolizumab (keytruda): Advanced or metastatic urothelial carcinoma. (2017). Available online at: https://www.accessdata.fda.gov/drugsatfda_docs/label/2017/125514s017s018lbl.pdf (Accessed May 9, 2017).

[14] Administration USFaD. Fda approves new, targeted treatment for bladder cancer. Silver spring, md: U.S: Food and drug administration (2016). Available at: http://www.Fda.Gov/newsevents/newsroom/pressannouncements/ucm501762.Htm (Accessed May 19, 2016).

[15] Luo J Martucci VL Quandt Z Groha S Murray MH Lovly CM. Immunotherapy-mediated thyroid dysfunction: Genetic risk and impact on outcomes with pd-1 blockade in non-small cell lung cancer. Clin Cancer Res. (2021) 27:5131–40. doi: 10.1158/1078-0432.CCR-21-0921 PMC8815444 34244291

[16] Villanova M Tolaney SM Min L. Association between pembrolizumab-related thyroid adverse events and outcomes in early-stage triple-negative breast cancer patients. Endocr Relat Cancer. (2024) 31:e240120. doi: 10.1530/ERC-24-0120 39235349

[17] Ikemoto J Ishii Y Serikawa M Tsuboi T Tsushima K Nakamura S. Pembrolizumab-induced focal pancreatitis diagnosed by endoscopic ultrasound-guided fine-needle aspiration. Intern Med. (2022) 61:2463–9. doi: 10.2169/internalmedicine.8507-21 PMC9449604 35022344

