# Supplementary material for: Effectiveness and safety of pembrolizumab, nivolumab, and atezolizumab as adjuvant therapy for high-risk muscle-invasive urothelial carcinoma: an indirect comparison
Source: Front Oncol. 2025 Jan 23;14:1527540. doi: 10.3389/fonc.2024.1527540 (PMC11798953; doi:10.3389/fonc.2024.1527540)
Supplement: Supplementary File 1 — Risk of bias assessment according to the Cochrane Collaboration tool RoB2 v9. (A) Risk of bias graph that illustrates the proportions of results at low risk, some concerns or high risk; (B) Traffic light plot shows the domain and overall judgments study-by-study. [file DataSheet1.pdf]

a

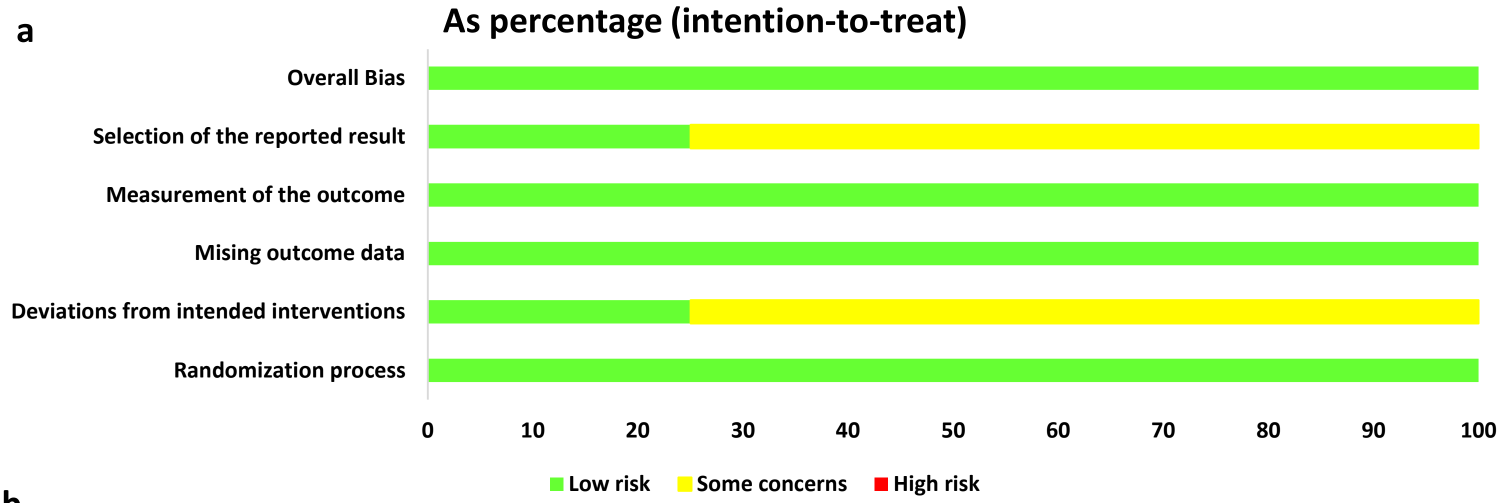

b

Clinical trial	Author	Experimental	Comparator	Outcome	Weight	D1	D2	D3	D4	D5	Overall
AMBASSADOR	Apolo	Pembrolizumab	Observation	DFS, OS	1	+	!	+	+	!	+
CheckMate 274	Bajorin	Nivolumab	Placebo	DFS	1	+	+	+	+	+	+
IMvigor010	Bellmunt	Atezolizumab	Observation	DFS	1	+	!	+	+	!	+
IMvigor010	Powles	Atezolizumab	Observation	OS	1	+	!	+	+	!	+

+ Low risk

! Some concerns

- High risk

D1 Randomisation process; D2 Deviations from the intended interventions; D3 Missing outcome data; D4 Measurement of the outcome; D5 Selection of the reported result
