# Supplementary material for: Effectiveness and safety of pembrolizumab, nivolumab, and atezolizumab as adjuvant therapy for high-risk muscle-invasive urothelial carcinoma: an indirect comparison
Source: Front Oncol. 2025 Jan 23;14:1527540. doi: 10.3389/fonc.2024.1527540 (PMC11798953; doi:10.3389/fonc.2024.1527540)
Supplement: Supplementary file 2 [file DataSheet2.pdf]

Supplementary file 2

Reconstructed individual participant data validation

Quantitative validation of reconstructed data

Trial	Treatment	Outcome	Original HR (95% CI)	Reconstructed HR (95% CI)	Z value	p value
IMvigor 010	Atezolizumab vs Observation	DFS	0.89 (0.74-1.08)	0.93 (0.77-1.12)	-0.16	0.87
IMvigor 010	Atezolizumab vs Observation	DFS (PD-L1+)	1.01 (0.75-1.35)	1.03 (0.77-1.39)	-0.05	0.96
IMvigor 010	Atezolizumab vs Observation	OS	0.91 (0.73-1.13)	0.94 (0.76-1.16)	-0.10	0.92
AMBASSADOR	Pembrolizumab vs Observation	DFS	0.73 (0.59-0.90)	0.75 (0.61-0.91)	-0.09	0.93
AMBASSADOR	Pembrolizumab vs Observation	DFS (PD-L1+)	0.81 (0.61-1.08)	0.79 (0.60-1.06)	0.06	0.95
AMBASSADOR	Pembrolizumab vs Observation	OS	0.98 (0.76-1.26)	0.95 (0.73-1.22)	0.08	0.93
CheckMate 274	Nivolumab vs Placebo	DFS	0.71 (0.58-0.86)	0.71 (0.59-0.86)	0.00	1.00
CheckMate 274	Nivolumab vs Placebo	DFS (PD-L1+)	0.52 (0.37-0.72)	0.53 (0.38-0.73)	-0.04	0.97
CheckMate 274	Nivolumab vs Placebo	OS	0.76 (0.61-0.96)	0.77 (0.61-0.96)	-0.04	0.97

DFS of Atezolizumab

Original Curve

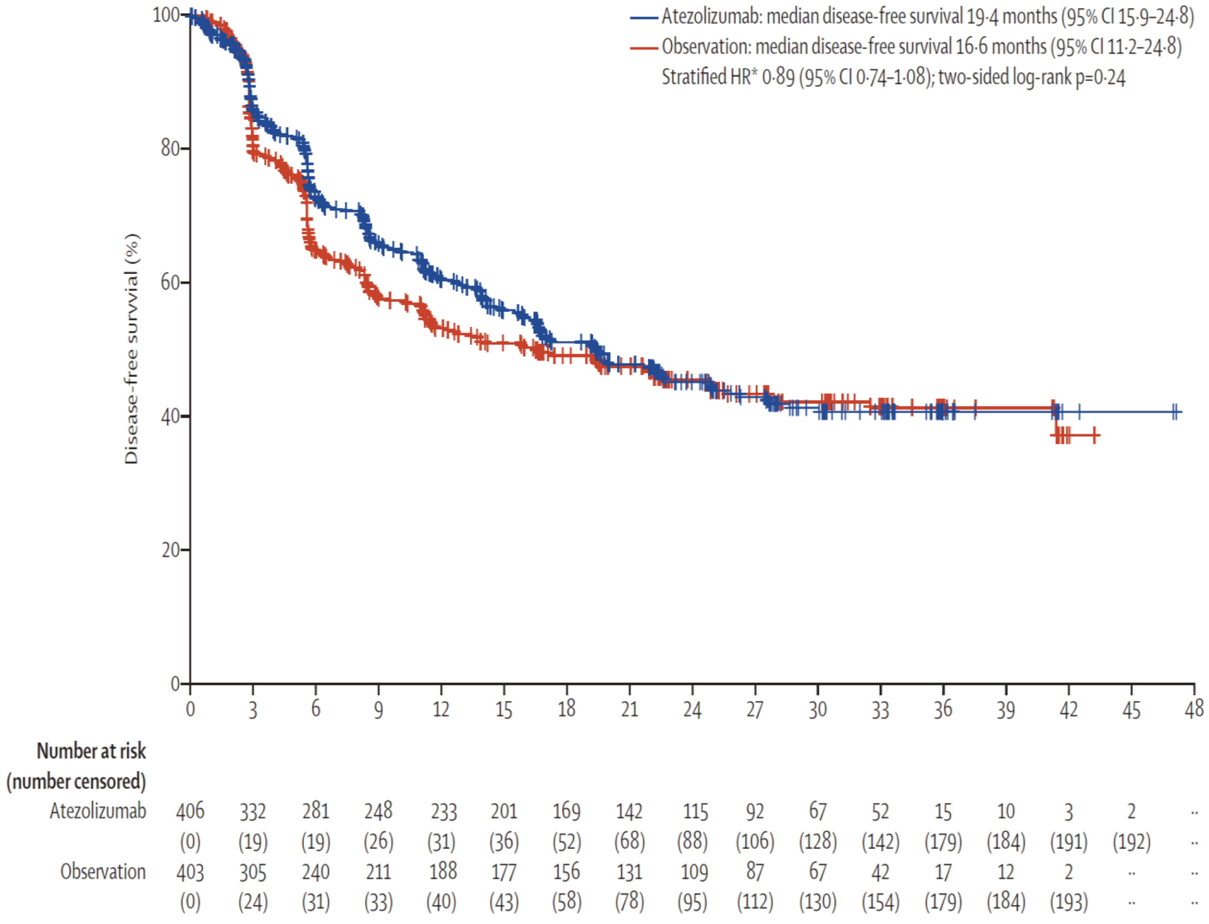

Reconstructed curve

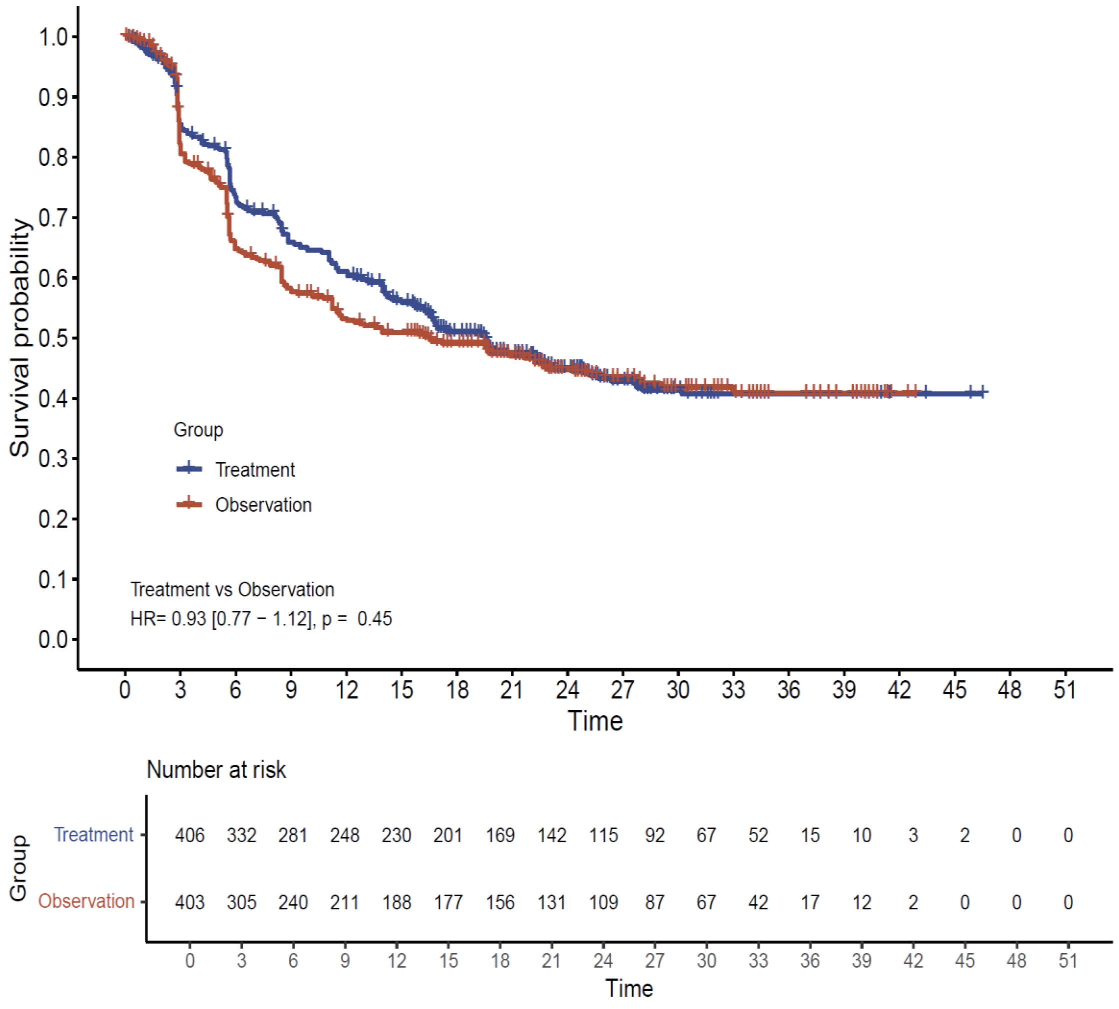

Bellmunt J, et al. Lancet Oncol. Apr 2021;22(4):525-537.

DFS of Atezolizumab (positive PD-L1 status)

Original Curve

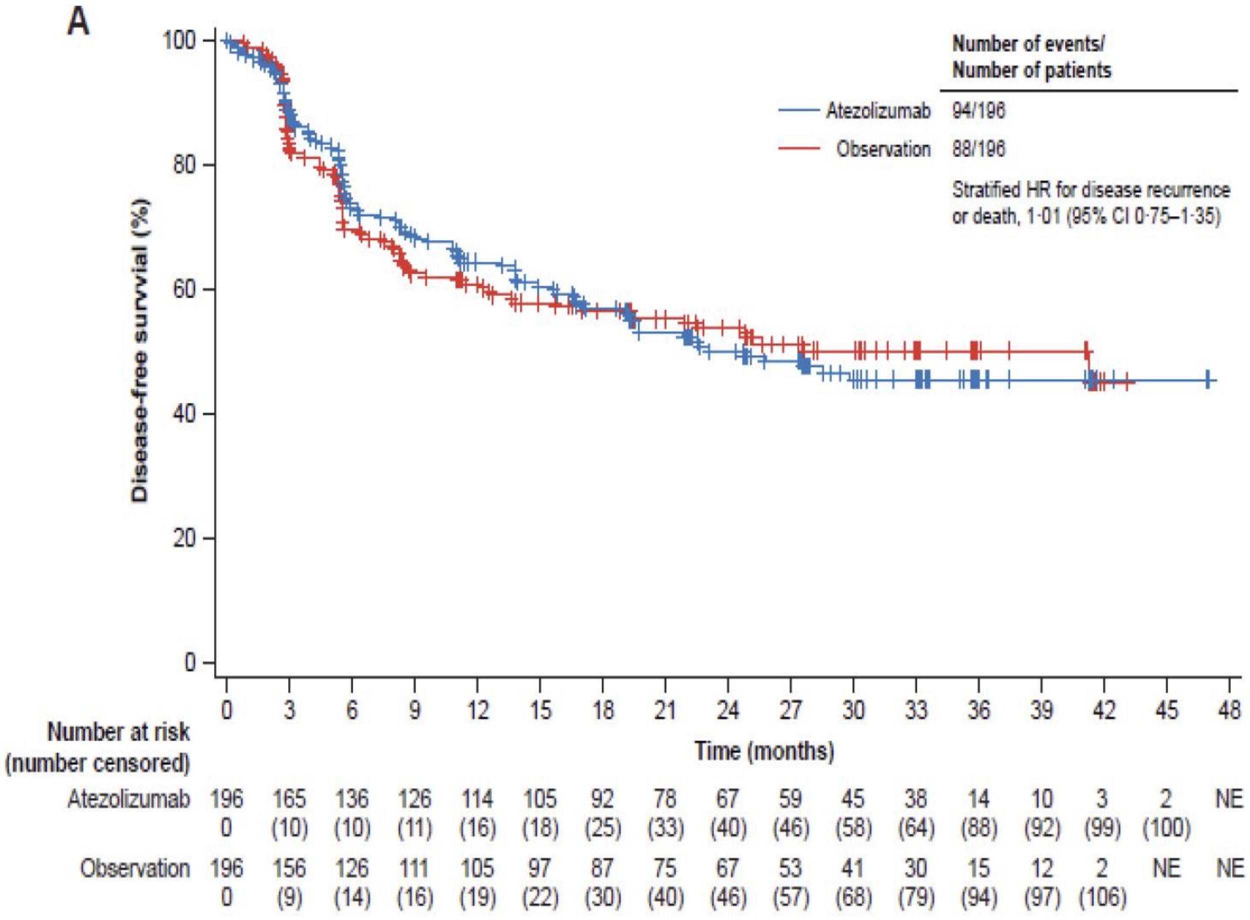

Reconstructed curve

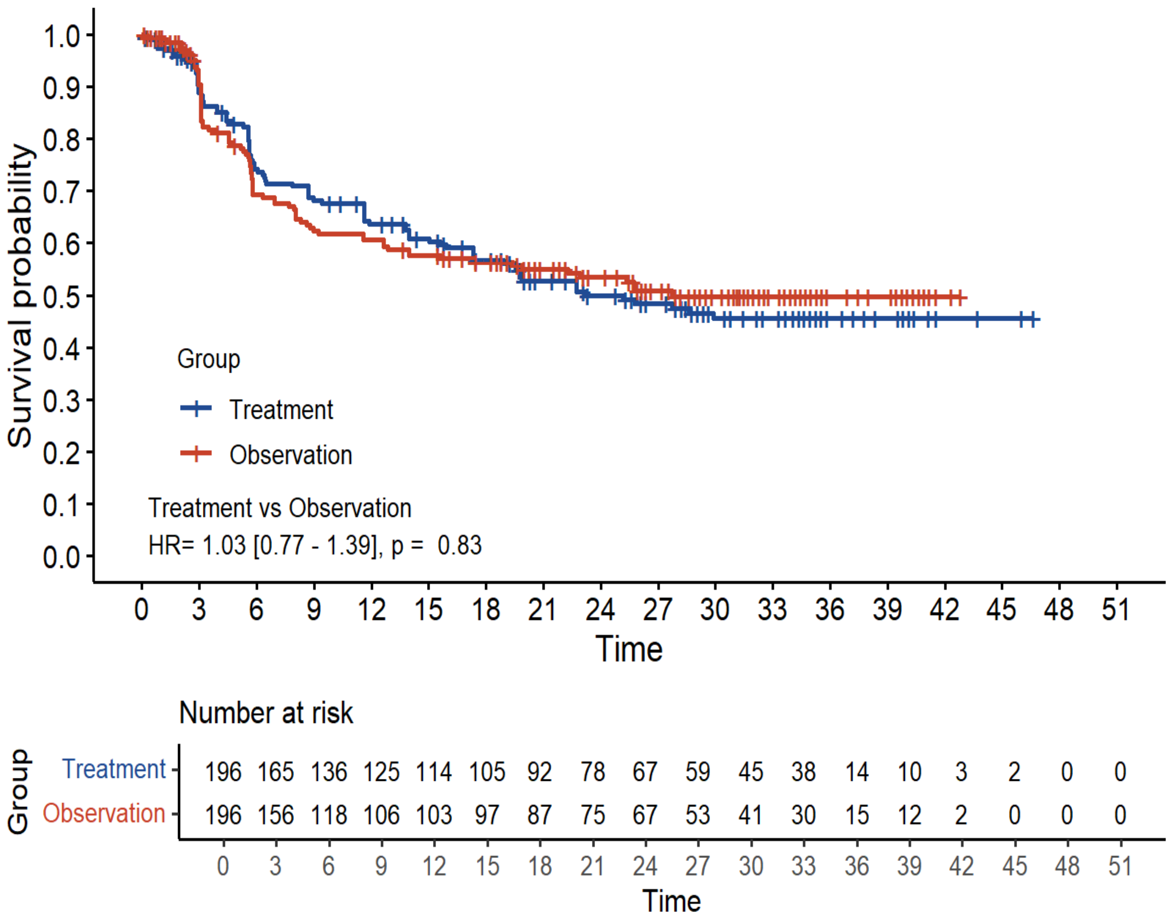

Bellmunt J, et al. Lancet Oncol. Apr 2021;22(4):525-537.

OS of Atezolizumab

Original Curve

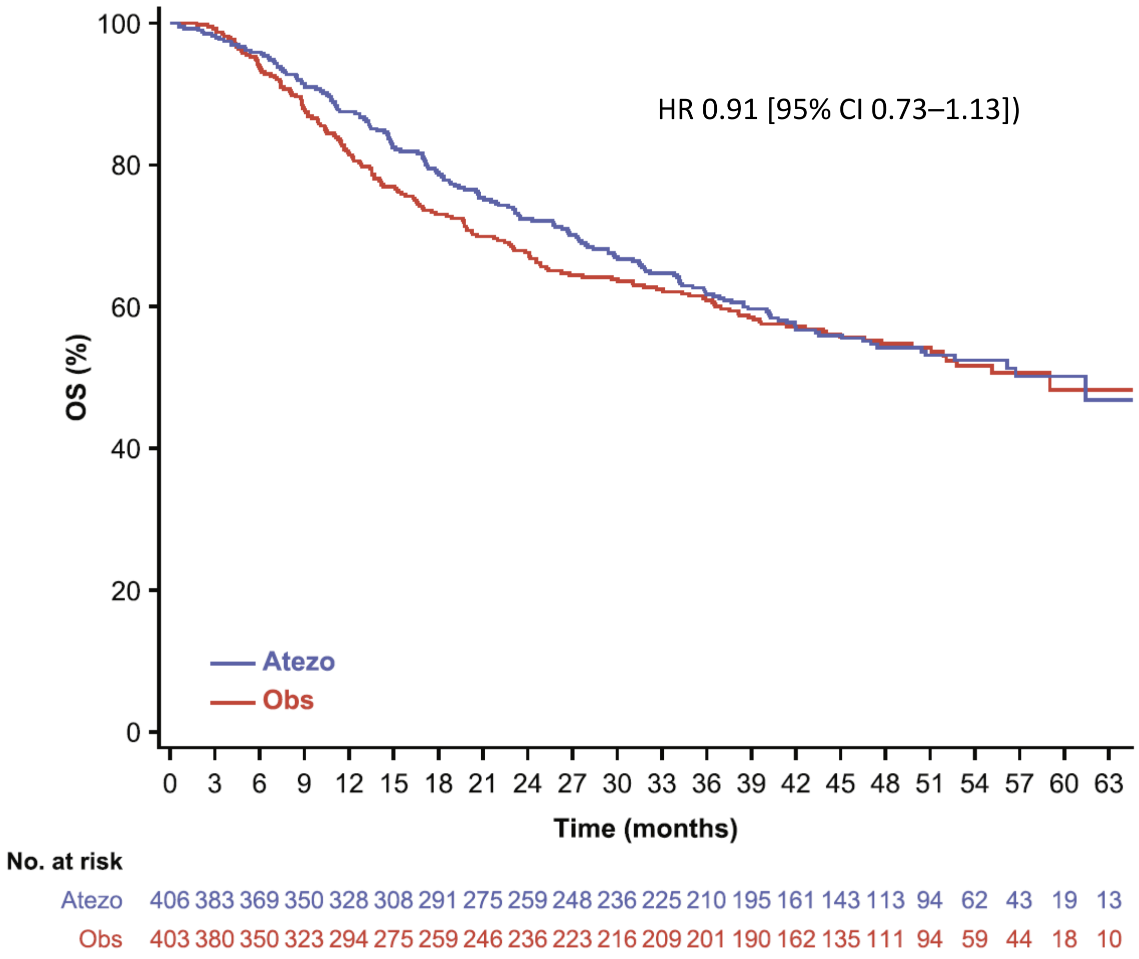

Reconstructed curve

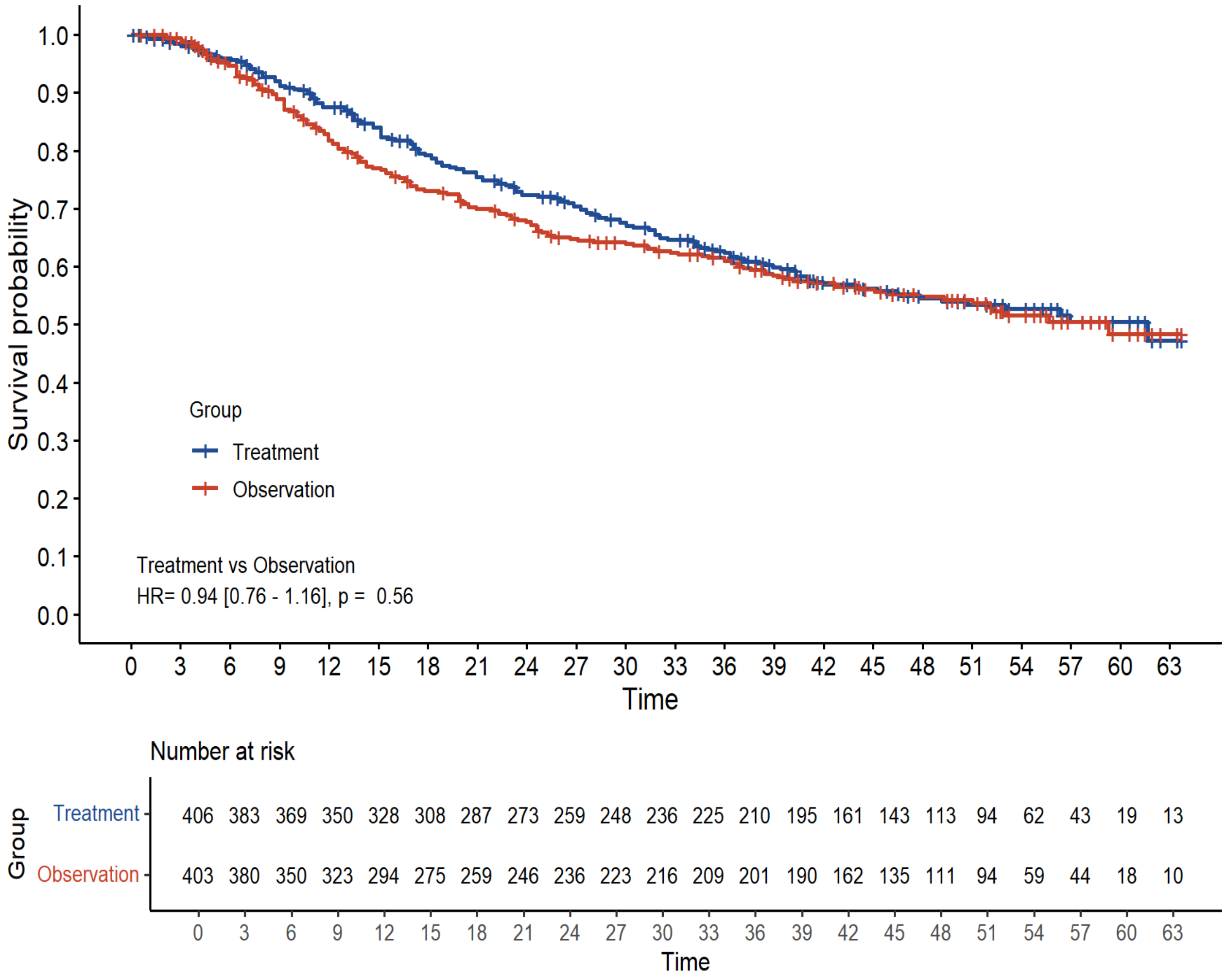

Powles T, et al. Eur Urol. 2024, 85(2): 114-122.

DFS of Nivolumab

Original Curve

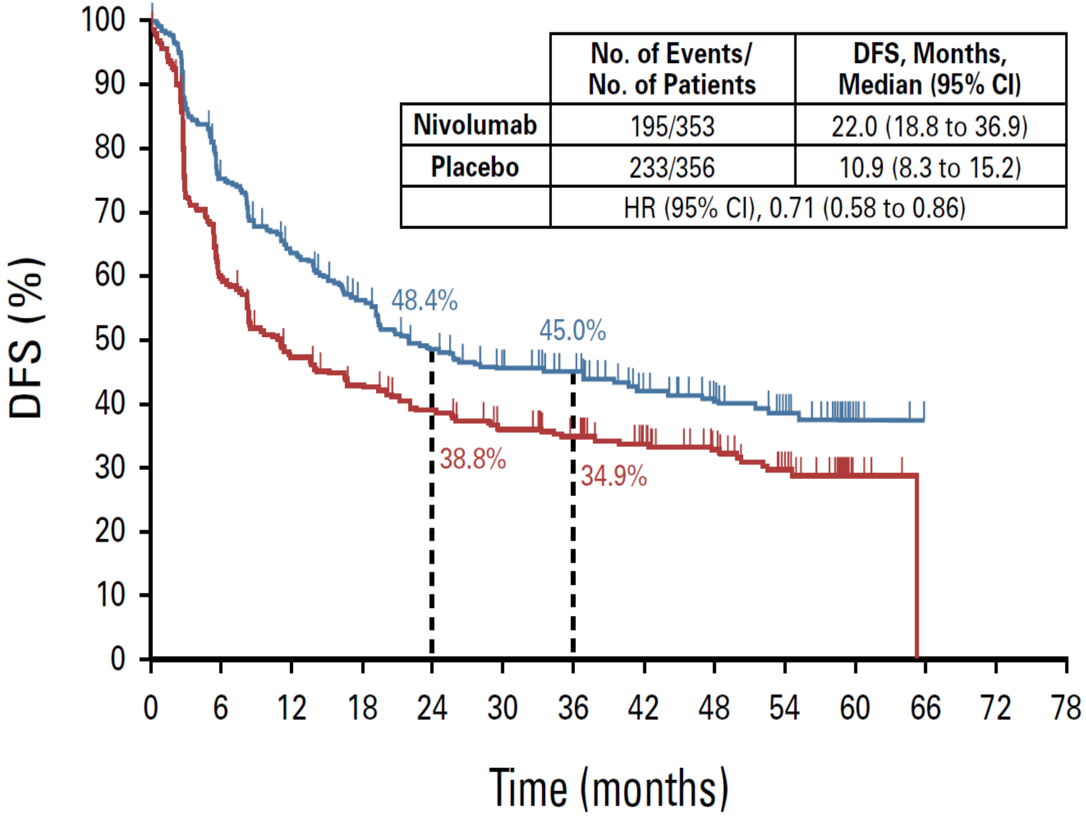

Number at risk

Nivolumab	353	253	208	177	150	132	113	83	57	43	4	0	0	0
Placebo	356	207	156	138	123	109	94	80	59	39	4	0	0	0

Reconstructed curve

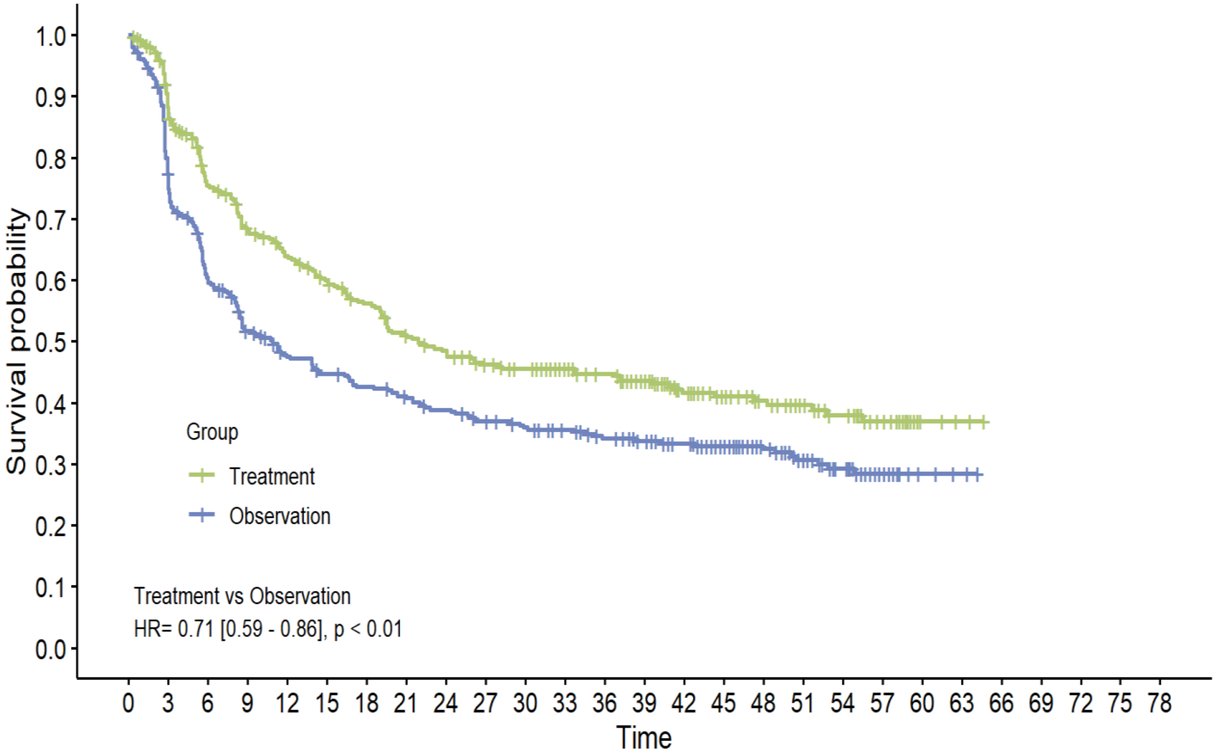

Number at risk

Group	0	3	6	9	12	15	18	21	24	27	30	33	36	39	42	45	48	51	54	57	60	63	66	69	72	75	78
Treatment	353	301	253	224	208	192	177	159	150	138	132	123	113	100	83	70	57	50	43	24	4	2	0	0	0	0	0
Observation	356	272	207	175	156	146	138	131	123	115	109	102	94	87	80	70	59	49	39	21	4	2	0	0	0	0	0

Time

Galsky MD, et al. J Clin Oncol. 2024: JCO2400340.

DFS of Nivolumab (positive PD-L1 status)

Original Curve

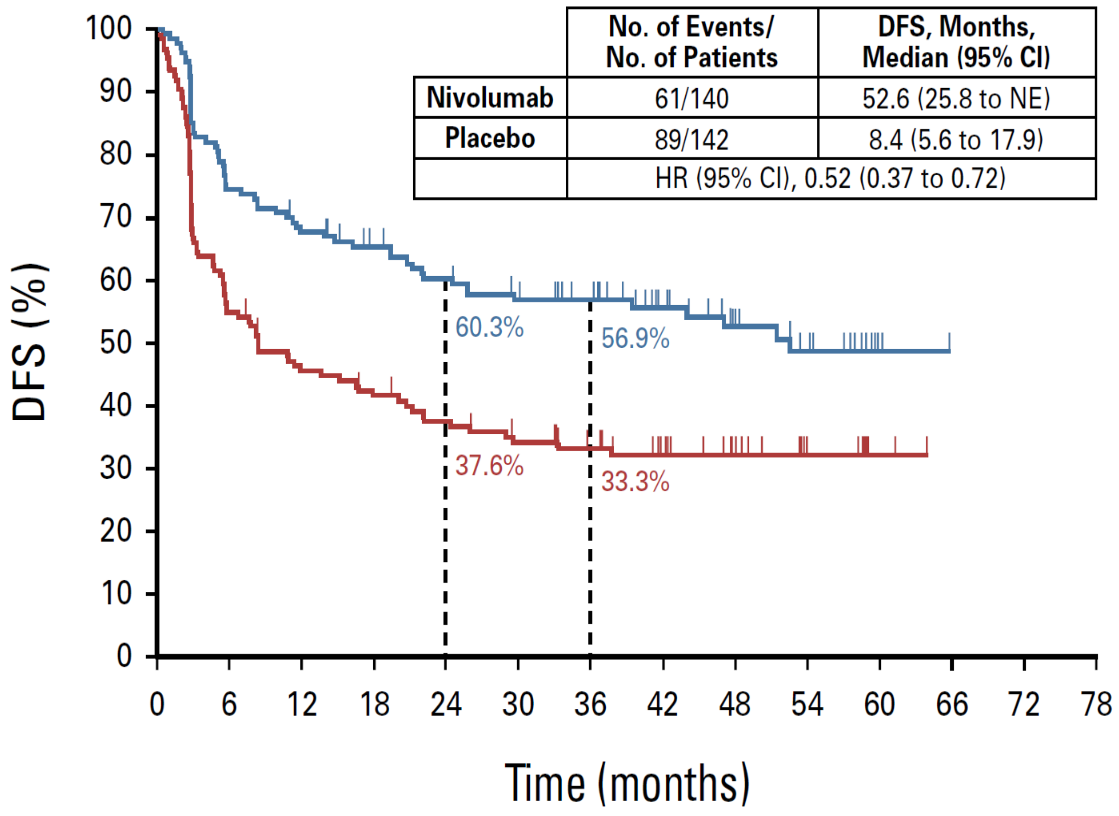

Number at risk

Nivolumab	140	99	88	79	72	64	55	42	29	23	2	0	0	0
Placebo	142	74	58	52	46	40	34	26	18	9	2	0	0	0

Reconstructed curve

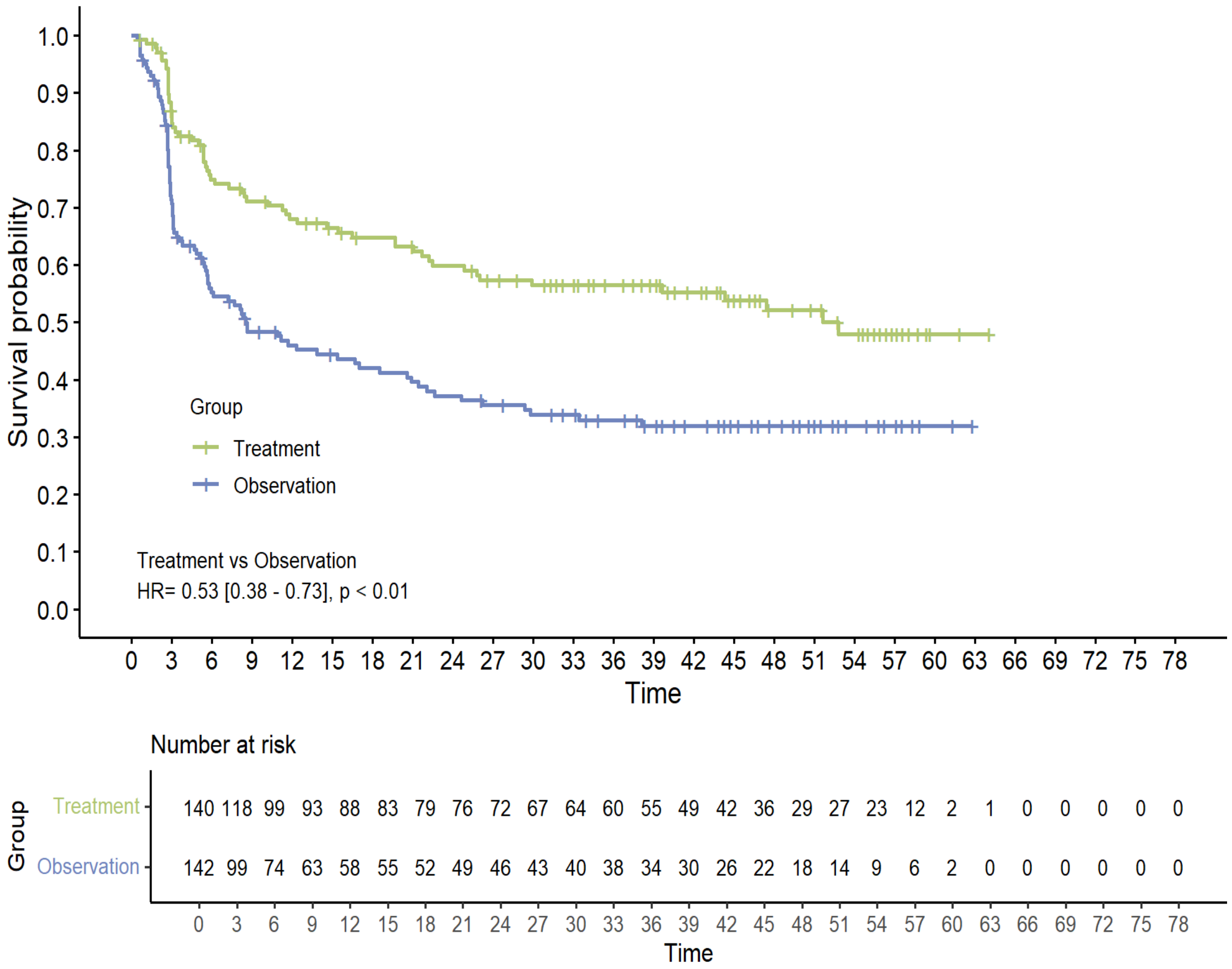

Galsky MD, et al. J Clin Oncol. 2024: JCO2400340.

OS of Nivolumab

Original Curve

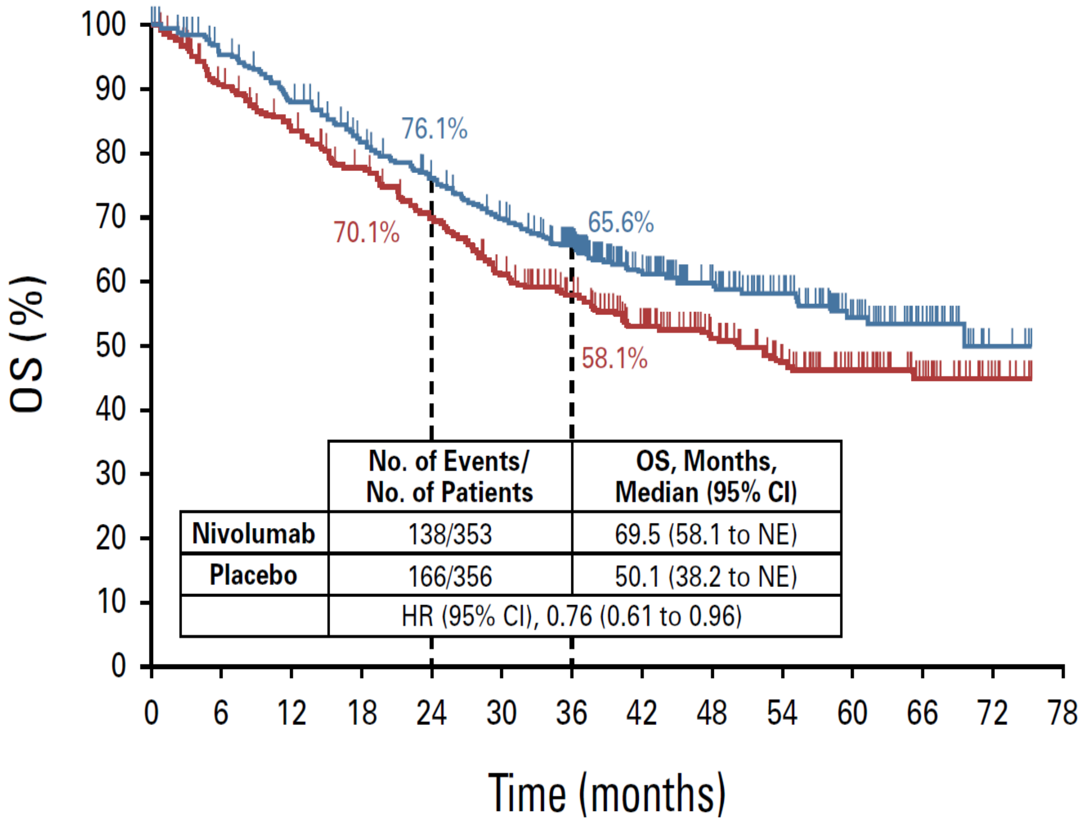

Number at risk

	0	6	12	18	24	30	36	42	48	54	60	66	72	78
Nivolumab	353	326	298	268	244	220	188	150	123	92	60	33	4	0
Placebo	356	308	281	254	226	194	167	136	109	79	56	32	10	0

Reconstructed curve

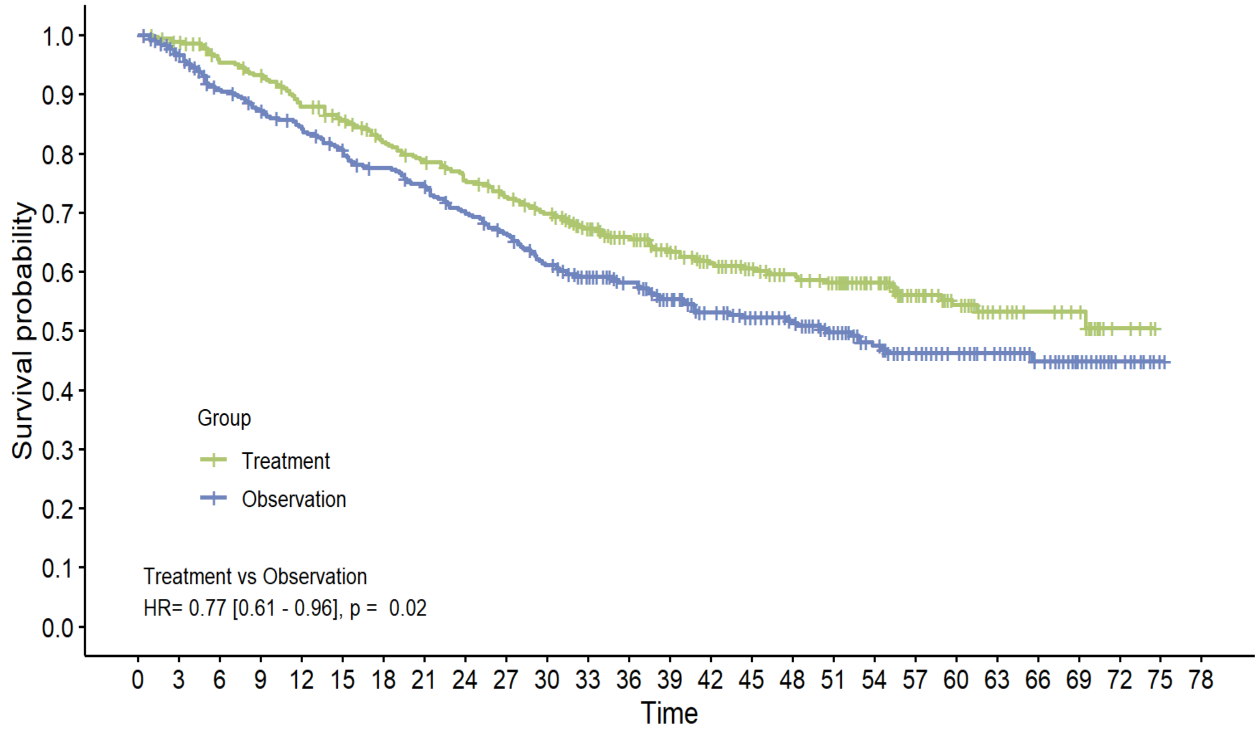

Number at risk

Group	0	3	6	9	12	15	18	21	24	27	30	33	36	39	42	45	48	51	54	57	60	63	66	69	72	75	78
Treatment	353	344	326	318	298	285	268	256	244	232	220	202	188	169	150	136	123	106	92	76	60	45	33	23	4	0	0
Observation	356	336	308	294	281	266	254	243	226	213	194	179	167	151	136	122	109	95	79	67	56	44	32	21	10	2	0

Time

Galsky MD, et al. J Clin Oncol. 2024: JCO2400340.

DFS of Pembrolizumab

Original Curve

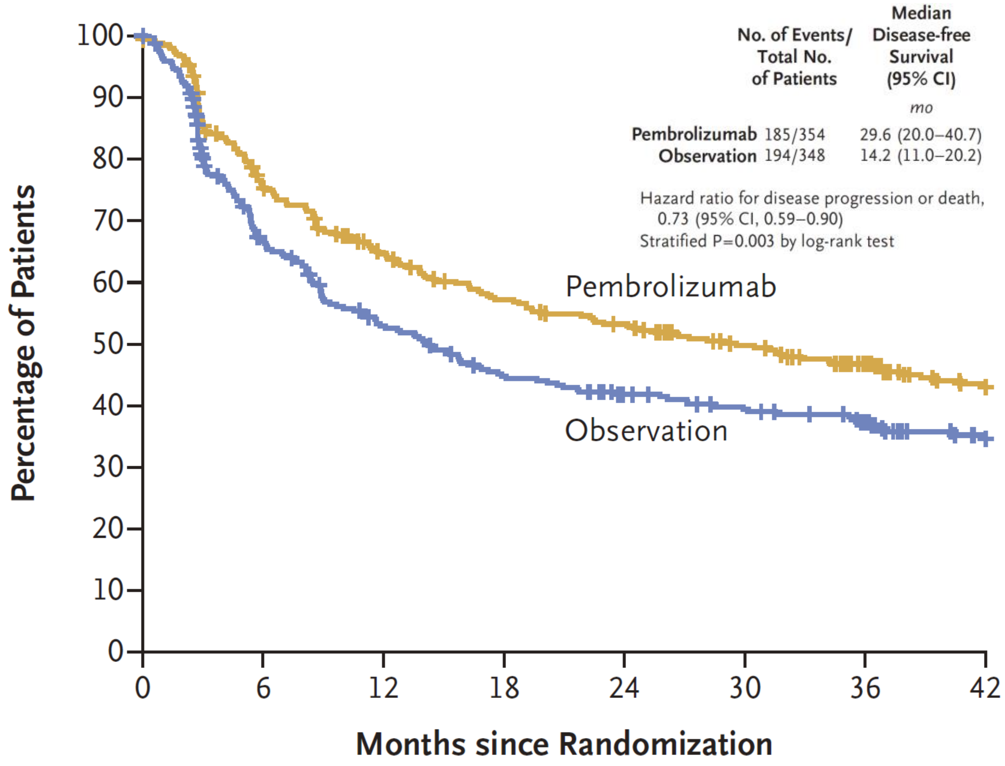

No. at Risk

Pembrolizumab	354	247	202	174	159	137	114	85
Observation	348	198	150	124	107	96	81	58

Reconstructed curve

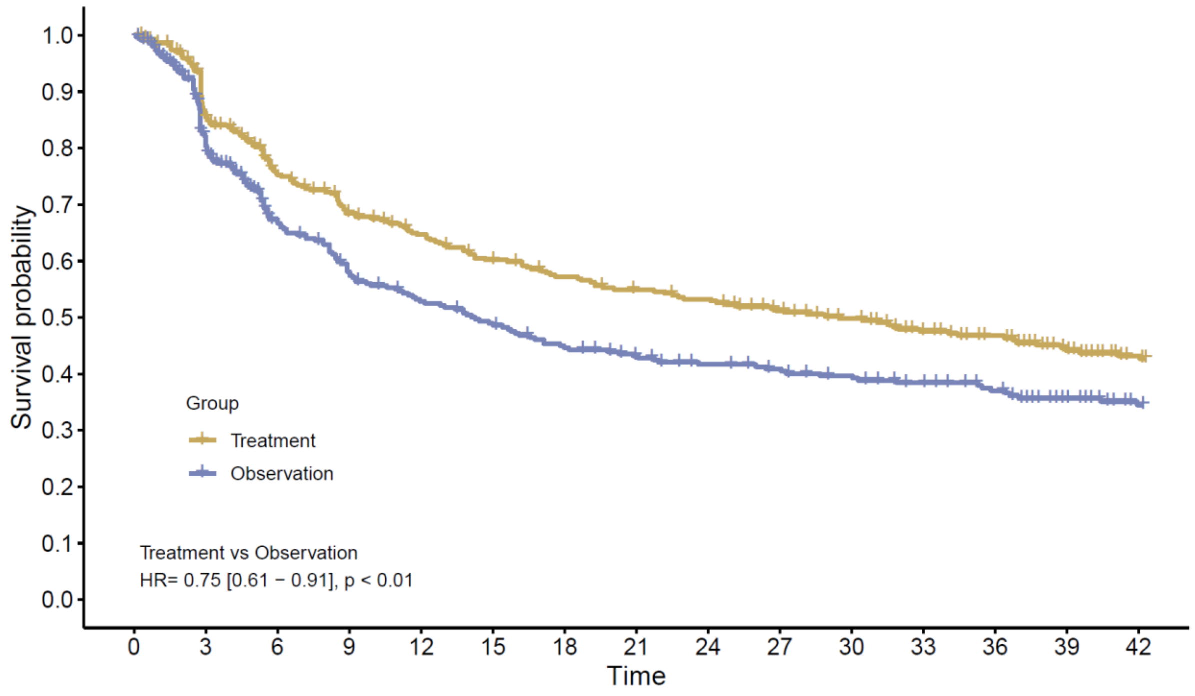

Number at risk

Group	0	3	6	9	12	15	18	21	24	27	30	33	36	39	42
Treatment	354	293	247	219	202	186	174	165	159	147	137	123	114	98	85
Observation	348	260	198	168	150	138	124	115	107	102	96	89	81	69	58

Time

Apolo AB, et al. N Engl J Med. Sep 15 2024. doi: 10.1056/NEJMe2411485.

DFS of Pembrolizumab (positive PD-L1 status)

Original Curve

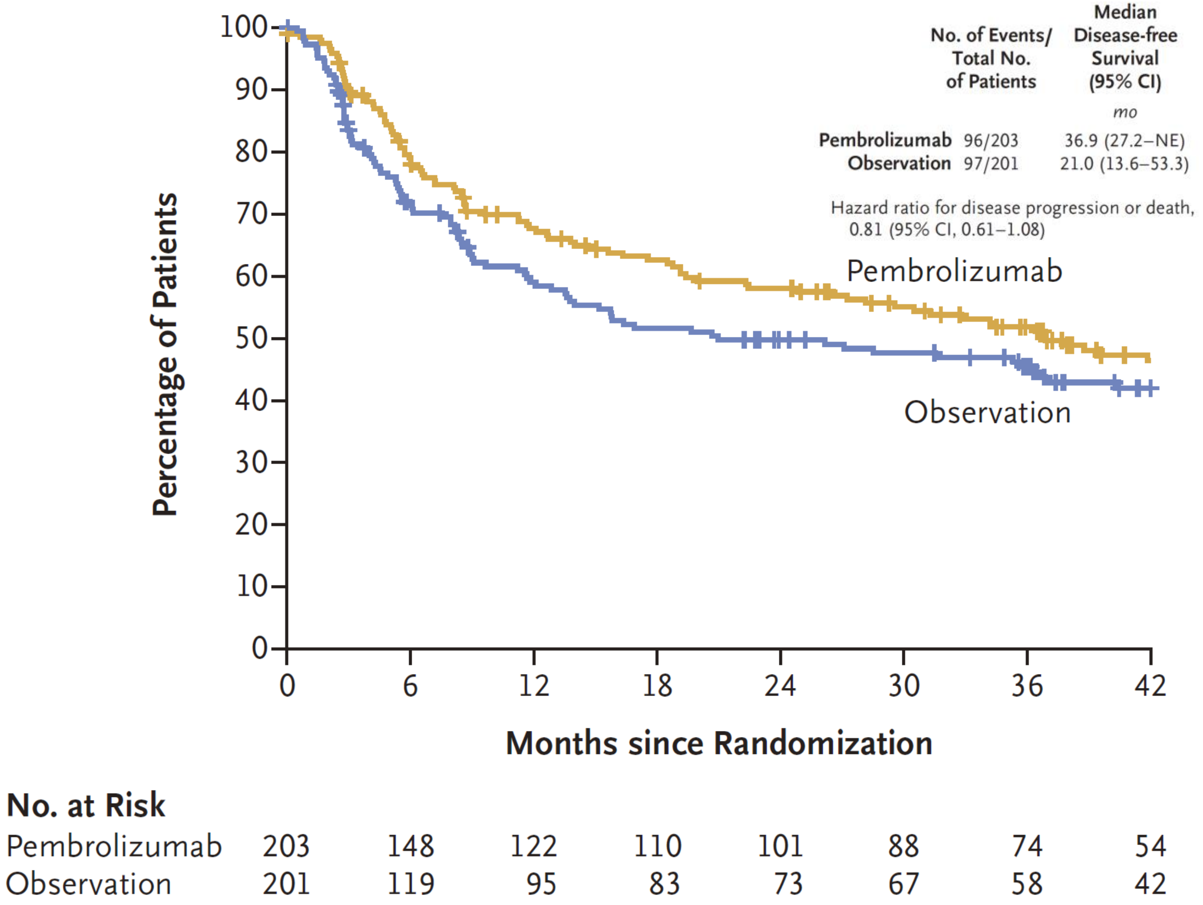

Reconstructed curve

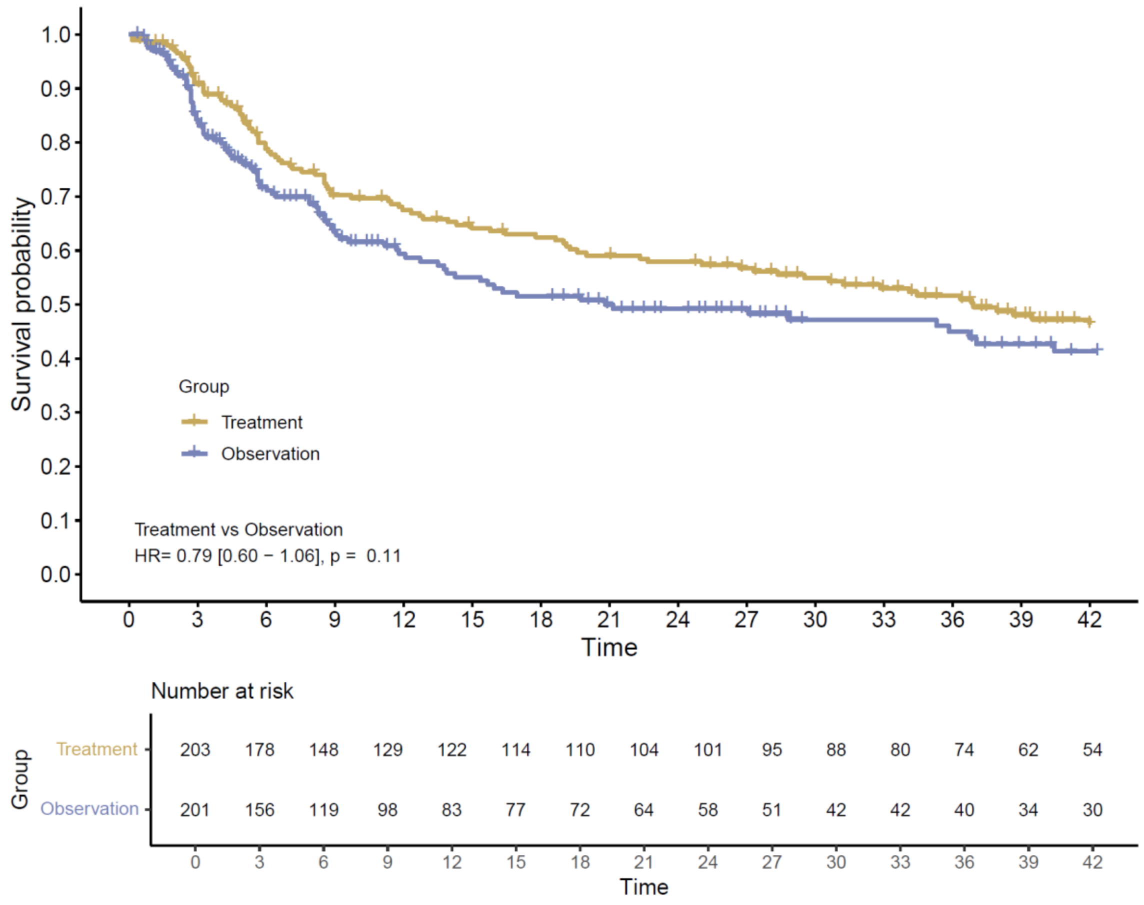

Apolo AB, et al. N Engl J Med. Sep 15 2024. doi: 10.1056/NEJMe2411485.

OS of Pembrolizumab

Original Curve

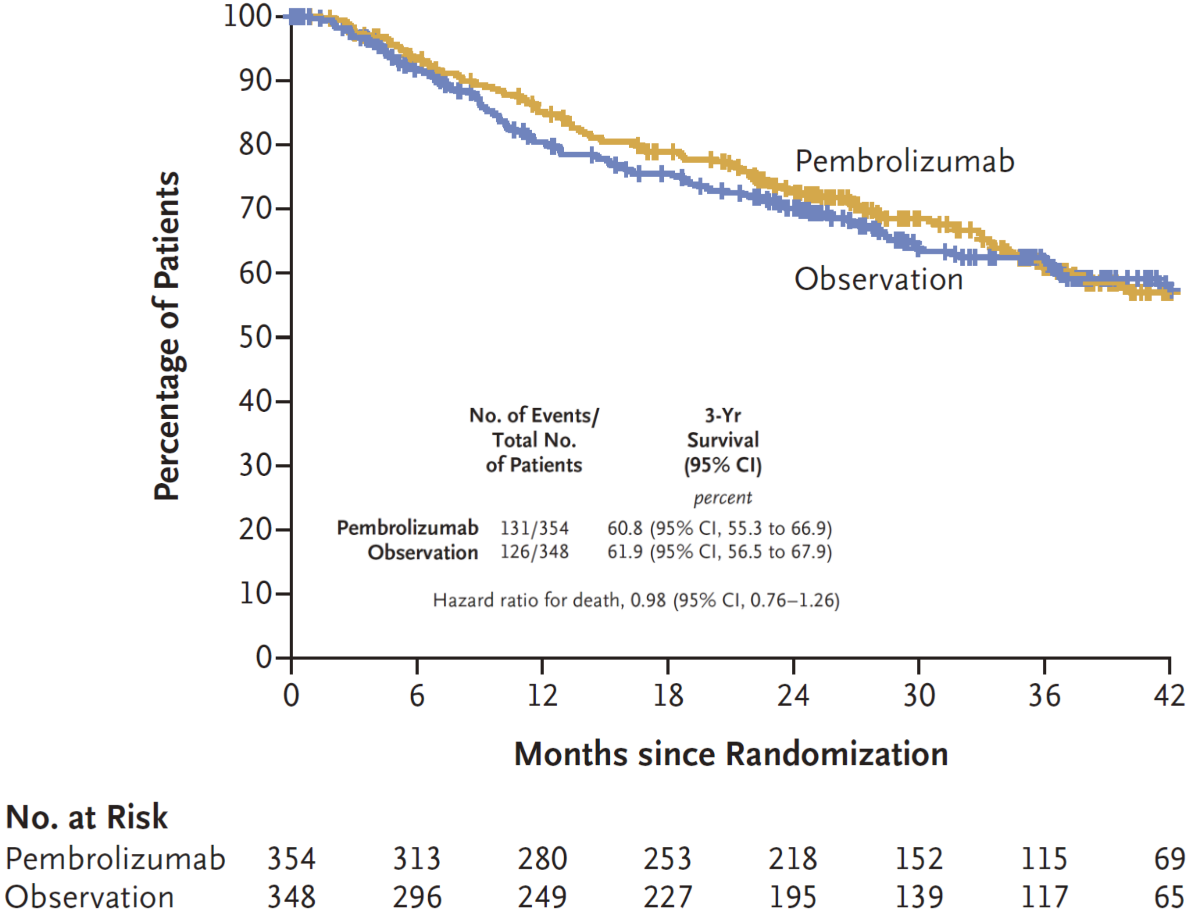

Reconstructed curve

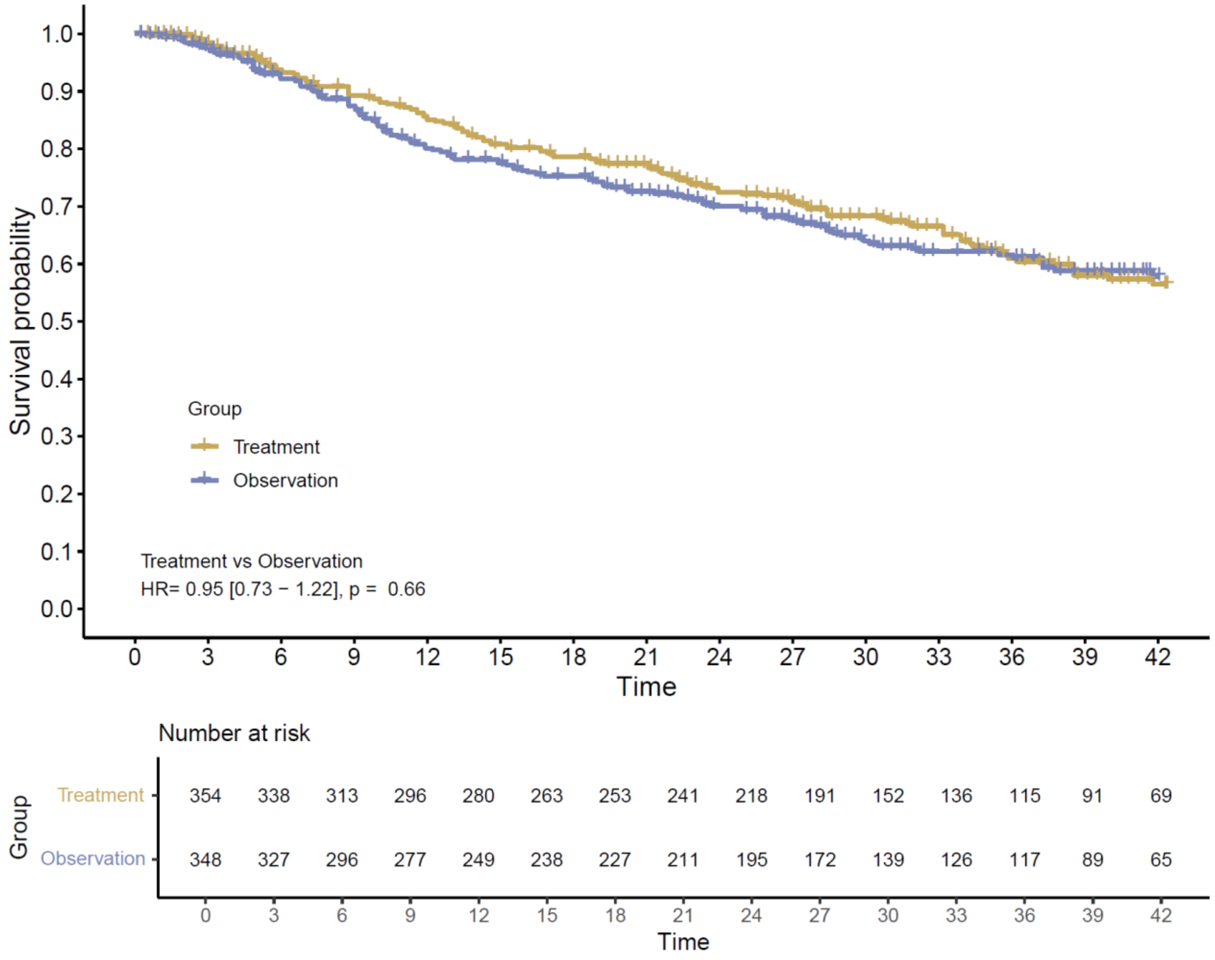

Apolo AB, et al. N Engl J Med. Sep 15 2024. doi: 10.1056/NEJMe2411485.
